# Genome-Wide Identification and Characterization of Amino Acid Polyamine Organocation Transporter Family Genes Reveal Their Role in Fecundity Regulation in a Brown Planthopper Species (*Nilaparvata lugens*)

**DOI:** 10.3389/fphys.2021.708639

**Published:** 2021-07-14

**Authors:** Lei Yue, Ziying Guan, Mingzhao Zhong, Luyao Zhao, Rui Pang, Kai Liu

**Affiliations:** ^1^Innovative Institute for Plant Health, College of Agriculture and Biology, Zhongkai University of Agriculture and Engineering, Guangzhou, China; ^2^Guangdong Provincial Key Laboratory of Microbial Safety and Health, State Key Laboratory of Applied Microbiology Southern China, Institute of Microbiology, Guangdong Academy of Sciences, Guangzhou, China

**Keywords:** *Nilaparvata lugens*, amino acid transporters, amino acid/polyamine/organocation, gene family, fecundity

## Abstract

The brown planthopper (BPH), *Nilaparvata lugens* Stål (Hemiptera:Delphacidae), is one of the most destructive pests of rice worldwide. As a sap-feeding insect, the BPH is incapable of synthesizing several amino acids which are essential for normal growth and development. Therefore, the insects have to acquire these amino acids from dietary sources or their endosymbionts, in which amino acid transporters (AATs) play a crucial role by enabling the movement of amino acids into and out of insect cells. In this study, a common amino acid transporter gene family of amino acid/polyamine/organocation (APC) was identified in BPHs and analyzed. Based on a homology search and conserved functional domain recognition, 20 putative APC transporters were identified in the BPH genome. Molecular trait analysis showed that the verified BPH APC family members were highly variable in protein features, conserved motif distribution patterns, and exon/intron organization. Phylogenetic analysis of five hemipteran species revealed an evolutionary pattern of interfamily conservation and lineage-specific expansion of this gene family. Moreover, stage- and tissue-specific expression analysis revealed diverse expression patterns in the 20 BPH APC transporter genes. Lastly, a potential BPH fecundity regulatory gene of *NlAPC09* was identified and shown to participate in the fecundity regulation through the use of quantitative polymerase chain reaction (qPCR) and RNA inference experiments. Our results provide a basis for further functional investigations of APC transporters in BPH.

## Introduction

As the key nutrients in animals, amino acid plays some irreplaceable roles in diverse biological processes, including energy regulating, protein synthesis, stress adaptation, nerve transmission, and cell development (Gordon, 1968; Wu, 2009; Wu et al., 2014). However, most insects do not have the ability to synthesize a group of 10 essential amino acids (histidine, isoleucine, leucine, lysine, methionine, phenylalanine, threonine, tryptophan, valine, and arginine) by themselves (Price et al., 2011). Consequently, they have to acquire these AAs from dietary sources or their endosymbionts (Ayayee et al., 2016; Zhao and Nabity, 2017). Cellular transport of amino acids is mediated by specific amino acid transporters (AATs). In insects, two major amino acid transporter families have been reported, which are the amino acid auxin permease (AAAP) family and the amino acid polyamine organocation (APC) family (Attardo et al., 2006; Price et al., 2011; Dahan et al., 2015). Both AATs family proteins showed great variations in length. AAAP family proteins contain 400–500 amino acid residues and possess 10–12 predicted transmembrane α-helical spanners (TMSs) (Chang and Bush, 1997; Young et al., 1999), while the length in APC family proteins range between 350–800 amino acid residues and are deduced to include 10–14 TMSs (Jack et al., 2000). A broad spectrum of substrates could be transported by AATs across the plasma membrane, including amino acids, nucleotides, peptides, and inorganic anions or cations (Wolfersberger, 2000). Previous studies have revealed that AATs could function as symporter and antiporters to ensure the proper cross-membrane flux of solutes (Wolfersberger, 2000).

Amino acids and the AATs have been reported to be vital in regulating insect reproduction (Carpenter et al., 2012; Smykal and Raikhel, 2015). During reproduction, insects require a massive input of nutrients from food sources for egg development (Terashima and Bownes, 2004; Attardo et al., 2005). The regulation of amino acid/target-of-rapamycin (AA/TOR) plays an important role in supplementing nutrients for egg development, with the corresponding pathway being well-maintained in insects (Roy and Raikhel, 2011; Smykal and Raikhel, 2015; Zhu et al., 2020). As a sensor of cellular nutritional status, the AA/TOR could activate the secretion of insect insulin-like peptides (ILPs), which interact with the insulin pathway to modify the titers of the juvenile hormone (JH) and ecdysone, which control the reproduction process of insect females, involving yolk protein production (vitellogenesis) and egg maturation (Smykal and Raikhel, 2015). Uptake of massive amounts of amino acids associated with vitellogenesis requires an effective transport system that facilitates the transport of amino acids across the plasma membrane. Knockdown of AATs, especially APC, could significantly inhibit the expression of vitellogenin genes in *Aedes aegypti*, which is similar to the regulatory effects of the TOR signaling pathway on insect reproduction (Attardo et al., 2006; Carpenter et al., 2012). However, to date, there have been very few studies investigating the involvement of AATs in insect reproduction.

The brown planthopper (BPH), *Nilaparvata lugens* (Stål) (Hemiptera: Delphacidae), is a notorious pest of rice, causing serious yield losses in Asian countries (Holt et al., 1996). The BPH is known for its robust reproductive capacity (Sun et al., 2015). The content of AAs in the diet has a significant influence on the reproduction of BPH (Pan et al., 2014). The regulation of fecundity mediated by amino acid uptake has been shown to activate the TOR signaling pathway in BPH (Zhai et al., 2015). The TOR pathway further induces JH biosynthesis, which regulates AA-mediated vitellogenesis in this insect (Lu et al., 2016). All the metabolic processes above involve AA transportation. However, the role of AA transporters in the reproduction of BPH is largely unknown because of the lack of a genome-wide identification and functional report on AA transporters in this species.

The first draft genome of BPH was reported in 2014 but consisted of large gaps (Xue et al., 2014). Nevertheless, a new genome has recently been reported, which shows a significant improvement to its assembly at the chromosomal level (Ma et al., 2021). Consequently, in this study, we first identified and characterized the APC family genes based on the new version of the genome sequence of BPH. The conserved protein motifs, gene structures, phylogenetic relationships, and expression profiles were analyzed for these verified APC members. Lastly, an APC transporter gene of adult- and ovary-specific high expression was identified and its function in the regulation of reproduction in BPHs was determined using quantitative polymerase chain reaction (qPCR) and RNA inference (RNAi) technologies.

## Materials and Methods

### Insect Strains

The BPH population used in gene cloning of APC transporters and RNAi experiments was originally collected in the Guangdong Province of China in 2012, and the insects were reared on rice seedlings of the Huanghuazhan cultivar in a greenhouse maintained at 26 ± 2°C, with a relative humidity (RH) of between 70 and 90%, and a photoperiod of 16 h:8 h (light: dark). The two genotypes of BPHs with different levels of fecundity, which were used in the qPCR experiment, were obtained from Sun Yat-sen University, Guangzhou, China, and were housed as previously reported by Liu et al. (2020).

### Identification of APC Transporter Genes in the BPH Genome

The APC amino acid transporters in BPHs were determined according to a previously described method by Xia et al. (2017). Briefly, the approaches of homology search and conserved functional domain recognition were both employed to screen potential APC transporters in the BPH genome. First, the latest version of BPH genome data and the AAT protein sequence of reference species (*Drosophila melanogaster* and *Acyrthosiphon pisum*) were obtained from the InsectBase^1^ and the NCBI database (GenBank assembly accession: GCA_000757685.1)^2^, respectively. Next, the APC protein sequences from *D. melanogaster* and *A. pisum* were used as queries to conduct BLASTP searches against BPH proteins with an E-value cut-off value of 1.0e-5. In parallel, we searched the BPH protein dataset using the hidden Markov model (HMM) file of an APC transporter (*AA_permease*: PF00324) downloaded from the Pfam database^3^ using HMMER v.3.01 software, with the expected cutoff E-value of <1.0e-10. Subsequently, all hits from both approaches were merged and redundancy was removed, rusting in the BPH APC transporter candidates. To ensure the accuracy of our results, the protein sequence of each putative APC transporter was retrieved from the Pfam website to verify the presence of an APC-specific functional domain. Furthermore, the candidate BPH APC transporters were verified by searching the BLASTP algorithm against the non-redundant protein (nr) database (see text footnote 2) to exclude redundant transcript fragments, faulty annotated sequences and genes potentially from endosymbionts (Meng et al., 2020; Wu et al., 2020). The resulting APC transporters were lastly renamed according to their respective locations on the BPH chromosomes.

With the same procedure, APC transporters from three other hemipteran species were also predicted, *viz*. *Sogatella furcifera*, *Diaphorina citri*, and *Diuraphis noxia*. The protein sequences of APC transporters from all four insect species are listed in Supplementary Table 1.

### Protein Characteristics and Phylogenetic Analysis of APC Transporters

For protein characteristic analysis, the relative molecular mass and theoretical isoelectric point for each verified APC transporter protein sequence from BPHs was first calculated using the ExPASy online tool^4^. Following this, the secondary protein structure and the transmembrane structure contained by each BPH APC transporter were further predicted by submitting corresponding protein sequences to the SWISS-MODEL^5^ and TMHMM^6^ websites, respectively. In addition, other information for BPH APC transporters, such as the gene loci on the chromosome and the number of exons, were further extracted from genome annotation documents (gff3 file) of BPHs.

The global alignment of amino acid sequences of all verified APC transporters from BPHs was implemented using the ClustalW v2.1 online tool^7^ at default parameters. The resulting aln file was then uploaded to the ESPript v3.0 website^8^ to display the final alignment results.

For phylogenetic analysis of these BPH APC members, the full-length amino acid sequences were first aligned using Muscle v3.8.31, and a maximum likelihood (ML) phylogenetic tree comprising 20 BPH APC transporters was conducted with IQ-Tree v2.0, using the best-fit nucleotide substitution models that were was determined by Model Finder implemented in this software according to Akaike information criteria (Kalyaanamoorthy et al., 2017; Minh et al., 2020). An ultrafast bootstrap (UFB) of 1,000 replicates was used in the tree building to assess nodal support. The obtained phylogenetic tree was visualized using the Evolview v3.0 online tool^9^. Using the same method, we generated another unrooted ML tree that comprised of APC transporters from five hemipteran species (BPH, *S. furcifera*, *D. citri*, *D. noxia*, and *Bemisia tabaci*) belonging to four different families, in which the APC amino acid sequences of *B. tabaci* were obtained according to a method reported by Xia et al. (2017) while those of the other four insect species were predicted in this study. Similarly, the best-fit nucleotide substitution model used in the ML tree construction was inferred by IQ-Tree v2.0.

### Conserved Motif Prediction, Gene Structure Visualization, and Chromosome Location

Using a MEME v5.5.3 online program^10^ with the maximum number of motifs set at 10, the conserved protein motifs of the BPH APC transporter family were predicted, and the sequence logo of each recognized motif was generated and visualized using the MEME program and TBtools software (version 1.089), respectively. In addition, TBtools was employed to display the number, position, and boundaries of the exons, introns, and UTRs inside each BPH APC transporter gene, based on the related annotation information file. Furthermore, we used TBtools to map all APC transporters to their corresponding chromosomes and to depict their location distributions.

### Spatial and Temporal Expression Analysis of APC Transporters in BPHs

To explore the spatial-temporal expression pattern of each APC transporter in BPHs, the RNA-seq data at 15 developmental stages (egg-24 h, egg-48 h, egg-5 days, 1st-24 h, 1st-48 h, 2nd-24 h, 2nd-48 h, 3rd-24 h, 3rd-48 h, 4th-24 h, 4th-48 h, 5th-24 h, 5th-48 h, female adult-24 h, and female adult-72 h) and five tissues (head, salivary glands, integument, gut, and ovaries) were downloaded from the NCBI database (SRR13958477-SRR13958499)^11^. A pseudoalignment-based software, Kallisto v0.46.1, was used to quantify the transcript abundance in transcripts per million (TPM) counts for each identified gene (Denecke et al., 2020; Sheng et al., 2021). After the expression data of all APC transporter genes were obtained, two heatmaps illustrating the expression profiles of each APC member at different developmental stages and different tissues of the BPH were generated using TBtools.

### Ovary Tissue Preparation and RNA Extraction

To investigate the role of *NlAPC09* in regulating reproduction in BPHs, ovaries were dissected from insects with both high and low fecundity. First, 150 unmated BPH females (1-day-old) were collected and chilled on ice. The comatose insects were then placed in a Petri dish that had been brushed with a 0.65% NaCl water solution. Under binocular conditions, the samples were dissected in a droplet of saline solution with forceps, and the ovary tissues were treated with TRIzol Reagent (Magen, Guangzhou, China) before being placed on ice. A total of 50 dissected ovaries were randomly selected and pooled as biological replicates, and three independent replicates were performed in this study. Total RNA Kit II (Omega Bio-tek, Norcross, GA, United States) was used to extract total RNA from pooled ovary tissue according to the manufacturer’s instructions. The purity and quality of all RNA samples were assessed using the RNA 6000 Nano LabChip kit and Bioanalyzer 2100 (Agilent Technologies, Santa Clara, CA, United States).

### Quantitative PCR (qPCR) Analysis

Total RNA (1 μg) was used to synthesize first-strand cDNA using the PrimeScript^TM^ RT reagent kit (Takara Bio, Inc., Otsu, Shiga, Japan). The qPCR assays were performed using a Light Cycler 480 System (Roche Diagnostics, Basel, Switzerland) and the SYBR^®^, FAST Universal qPCR Kit (KAPA, Woburn, MA, United States) following the manufacturer’s instructions. A 10 μL reaction mixture containing 1 μL cDNA, 0.3 μL each of 10 μmol/L forward and reverse primers, and 5 μL SYBR^®^, FAST Universal qPCR mix (KAPA Biosystems, Woburn, MA, United States) was prepared. The PCR amplification conditions were as follows: 5 min at 95°C, followed by 45 cycles at 95°C for 10 s, 60°C for 20 s, and 72°C for 20 s. Three biological and three technical replicates for each sample were performed. Gene expression levels were normalized to the expression levels of BPH β*-actin* (Chen et al., 2013). Changes in gene expression were calculated using the 2^–△^^△^^Ct^ method (Livak and Schmittgen, 2001), and the results are expressed as mean ± SE. The differences in gene expression levels between treatments were analyzed using the Student’s *t*-test in the SPSS 18.0 statistical software (*P* < 0.05). In this study, the expression profile of *NlAPC09* in the ovaries of BPHs from two types of populations (described above) was determined, and the primers used for qPCR experiments are listed in Supplementary Table 1.

### Double-Stranded RNA (dsRNA) Synthesis and Injection Into BPHs

The DNA template for dsRNA synthesis was first amplified using primers containing the T7 promoter sequence at both ends. Next the purified DNA template was used to synthesize dsRNA of ds*NlAPC09* (502 bp) using the MEGAscript T7 Transcription Kit (Ambion, Austin, TX, United States) following the manufacturer’s protocol. Following this, we used a NanoDrop 2000 instrument (Thermo Fisher Scientific, Waltham, United States) to quantify the concentration of ds*NlAPC09*. Finally, the size and quality of ds*NlAPC09* were determined by electrophoresis on a 1% agarose gel, in which ds*GFP* was used as a control (Pang et al., 2017). The primers used for dsRNA synthesis are listed in Supplementary Table 1.

For dsRNA administration, 80 brachypterous female adults (1-day-old) were collected and anesthetized with carbon dioxide. Approximately 150 ng ds*NlAPC09* was then injected into 40 randomly selected insects before all samples/insects were maintained on a fresh rice plant growing in a transparent polycarbonate jar with a diameter of 15 cm and a height of 75 cm. The interference efficiency of ds*NlAPC09* was determined at 24, 48, and 72 h after dsRNA injection through RNA isolation and qPCR quantification on five insects at each time point. The remaining insects (approximately 20 individuals) were used for fecundity measurement as follows: ds*GFP* was used as a control, with the same procedure being followed as outlined above.

### Measurement of Fecundity in BPHs

Female adults injected with ds*NlAPC09* or ds*GFP* were paired with two untreated males and then transferred to a fresh rice plant as described above. More than 20 independent trials were performed for each treatment group. All insects were uniformly removed from rice plants 7 days later, and the newly hatched nymphs (<12 h) were recorded daily and removed until no nymphs hatched for 4 consecutive days. The total number of nymphs from each female was therefore obtained. Subsequently, we dissected all the rice plants using scalpels and microforces to count the unhatched eggs under a Leica dissecting microscope. The female fertility, i.e., the egg laying amount, was calculated as the sum of the nymph number and the unhatched eggs for each female adult. Lastly, we used the values of the nymph number divided by the egg laying amount as the hatching rate of BPH eggs.

### Statistical Analysis

The correlation analysis between the number of APC transporters on the same chromosome and the corresponding chromosome length was conducted using the Pearson’s test with SPSS 17.0 statistical software. For qPCR analysis, the relative mRNA expression level of *NlAPC09* was quantified using the 2^–Δ^ ^Δ^ ^CT^ method as described by Liu et al. (2020), in which the housekeeping gene, β*-actin*, was used to normalize the mRNA levels of the target gene (Chen et al., 2017). Student’s *t*-test was used to analyze the difference in the transcriptional level of *NlAPC09*, BPH fecundity (including total egg laying amount and nymph number), and the hatching rate of eggs deposited between different populations or treatments using SPSS 17.0. *P* < 0.05 were considered to be significant (marked ^∗^) while *P* < 0.01 were considered to be very significant (marked ^∗∗^). The results are presented as mean ± SEM.

## Results

### Identification and Characterization of APC Transporters in the BPH Species

**FIGURE 1:**
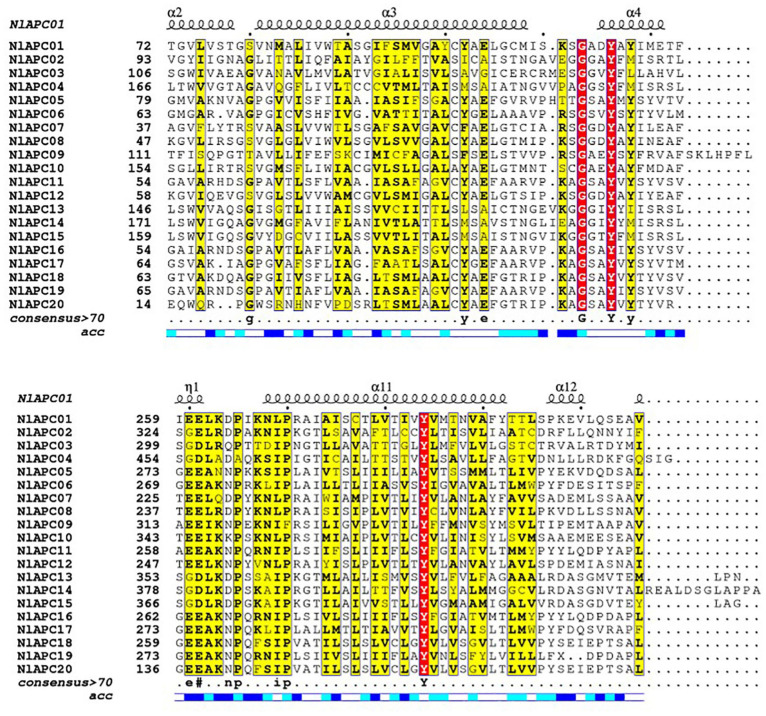
Amino acid sequence alignment and the secondary protein structure prediction for APC transporters from BPH. Amino acid sequences of all 20 BPH APC genes were aligned by ClustalW v2.1 and were displayed using ESPript v3.0. Secondary structure elements prediction for each APC gene was performed on the SWISS-MODEL website (https://swissmodel.expasy.org/), and the results of *NlAPC01* is shown above the alignment.

Twenty APC transporters with complete cDNA sequences were identified in the BPH genome and named *NlAPC01* to *NlAPC20* according to their position information on the chromosome (Table 1 and Supplementary Table 2). By searching for the conserved domains in the Pfam database, it was found that at least one of the three types of APC-featured domains, namely AA_permease, AA_permease_2, and AA_permease_C, were present in these genes (Supplementary Figure 1). The coding regions of these APC genes were verified using RT-PCR. The analysis results of protein features showed that the 20 transcripts of these APC transporters encoded between 222–1122 amino acids, with predicted theoretical isoelectric points (pIs) ranging from 6.13–8.66 and predicted molecular weights (MWs) ranging from 23.9 to 124.0 kDa (Table 1). Multiple alignment of amino acid sequences from all verified APC transporters indicated that although the protein sequences of APC transporters were highly variable in most regions, their N-terminals were comparatively conserved compared to the sequences located at the C-terminus (Figure 1). The results of protein structure analysis showed that multiple α-helices were contained in the secondary structure of APC transporters (Figure 1), and almost all APC transporters possessed more than 10 TMSs, except *NlAPC20*, which was predicted to have five TMSs (Table 1).

**TABLE 1:** Identification and characteristics of APC transporters in BPH.

Gene name	NCBI identifier	Genome identifier	Locus			CDS	exon	TMSs	Mw (kDa)	pI	Strand
								
			Chr.	starting	Ending						
*NlAPC01*	*XP_039277525*	Nlug07038-TA	chr1	20399983	20409472	506	3	12	55281.7	8.13	+
*NlAPC02*	*XP_039283066*	*Nlug21226-TA*	chr1	743456	774674	957	14	13	104777.1	7.81	+
*NlAPC03*	*XP_039278419*	*Nlug07271-TA*	chr2	65551982	65582822	677	13	11	73858.5	7.8	−
*NlAPC04*	*XP_039276024*	*Nlug07490-TA*	chr2	43317763	43363331	1079	23	10	119130.4	6.64	−
*NlAPC05*	*XP_039279370*	*Nlug01129-TA*	chr3	26888138	26912423	792	12	14	85532.8	6.13	+
*NlAPC06*	*XP_039279453*	*Nlug02200-TA*	chr3	31055051	31068378	510	7	11	55710.3	7.7	−
*NlAPC07*	*XP_022198118*	*Nlug06371-TA*	chr3	78249940	78273002	485	3	12	52475.7	7.33	−
*NlAPC08*	*XP_039281097*	*Nlug19179-TA*	chr3	95938510	95950515	494	4	11	53958.9	7.79	−
*NlAPC09*	*XP_039286832*	*Nlug06688-TA*	chr6	45746805	45772211	671	15	12	72853.2	7.43	+
*NlAPC10*	*XP_039288486*	*Nlug07560-TA*	chr7	31287561	31321892	586	10	12	63325.7	6.96	+
*NlAPC11*	*XP_039288773*	*Nlug13938-TA*	chr7	43966644	43979867	602	11	14	65792.0	8.66	+
*NlAPC12*	*XP_039290058*	*Nlug00063-TA*	chr8	17493916	17510991	495	12	12	54414.6	6.96	−
*NlAPC13*	*XP_039289667*	*Nlug11217-TA*	chr8	2510817	2535142	1062	13	11	117217.1	7.38	+
*NlAPC14*	*XP_039291870*	*Nlug19381-TA*	chr9	43515190	43580863	1098	20	13	121840.4	7.37	+
*NlAPC15*	*XP_039291803*	*Nlug22218-TA*	chr9	40431699	40509646	1122	24	11	124016.4	7.73	−
*NlAPC16*	*XP_039296825*	*Nlug17187-TA*	chr14	16084741	16128173	615	21	14	67035.3	8.49	+
*NlAPC17*	*XP_039296817*	*Nlug17189-TA*	chr14	15988763	16025229	611	13	15	66179.6	8.42	−
*NlAPC18*	*XP_039296923*	*Nlug20896-TA*	chr14	18470291	18500414	628	10	14	67808.4	6.34	−
*NlAPC19*	*XP_039296913*	*Nlug21374-TA*	chr14	17485099	17501514	475	8	10	51234.1	6.39	−
*NlAPC20*	*XP_039301531*	*Nlug28084-TA*	Sf7363	758	10624	222	6	3	23926.7	6.65	+

*TMSs, transmembrane structures; MW, Molecular weight; pI, Isoelectric point; +, the sense strand; -, the antisense strand.*

### Evolutionary Relationship, Conserved Motifs, and Gene Structure Analysis of BPH APC Genes

**FIGURE 2:**
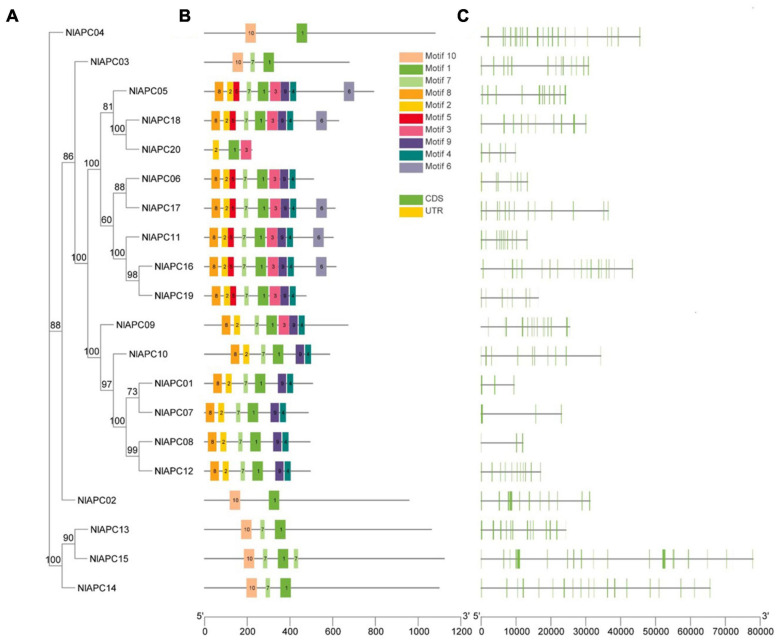
Phylogenetic relationships, motif compositions, and gene structures of identified APC transporter genes from BPH. **(A)** Phylogenetic tree of 20 APC gene family members identified in BPH. The ML phylogenetic tree was built using IQ-Tree software version 2.0 based on the best-fit model of LG + G4, and bootstrap values shown on branches were calculated based on 1,000 replicates. **(B)** Conserved motifs contained by each BPH APC transporter protein. The conserved motifs were predicted by MEME Suite 5.5.3 and were presented by the boxes of different colors. **(C)** The exon-intron structure of each identified BPH APC transporter gene analyzed by TBtools v1.089. Yellow boxes, green boxes and black lines represent non-coding regions, exons, and introns, respectively.

To survey the phylogenetic relationship among identified BPH APC transporters, a ML phylogenetic tree was constructed based on the best-fit model of LG + G4 using the IQ-Tree v2.0 software. Phylogenetic tree analysis showed that all 20 APC proteins could be categorized into three subgroups, which included 1, 16, and 3 members, respectively (Figure 2A).

To better understand the evolutionary conservation and possible duplication events within the BPH APC gene subfamily, the distribution pattern of conserved motifs and the organizational diversity of gene structures on all 20 APC transporters were investigated and visualized using TBtools (Figures 2A–C). The analysis results of the conserved motifs showed that a total of 10 conserved motifs were identified in BPH APC protein sequences, designated motifs 1–10, and their corresponding sequences were generated using the MEME program (Supplementary Figure 2). Among them, only motif 1 was present in all APC members; this might be the characteristic motif of the APC gene family (Figure 2B). Furthermore, the similarity in distribution patterns for conserved motifs was higher on the APC genes within the same subbranch of the phylogenetic tree but lower in those that were clustered into different subgroups (Figures 2A,B).

Gene structure analysis results showed that the number of exons within the BPH APC transporter gene family was highly variable, which ranged from 3 to 24 (Table 1 and Figure 2C). Furthermore, in contrast to conserved motifs, there was no obvious similarity in the exon/intron organization of the BPH APC genes from the same subgroup in the phylogenetic tree (Figures 2A,C).

### Intrachromosomal Distribution of BPH APC Genes

**FIGURE 3:**
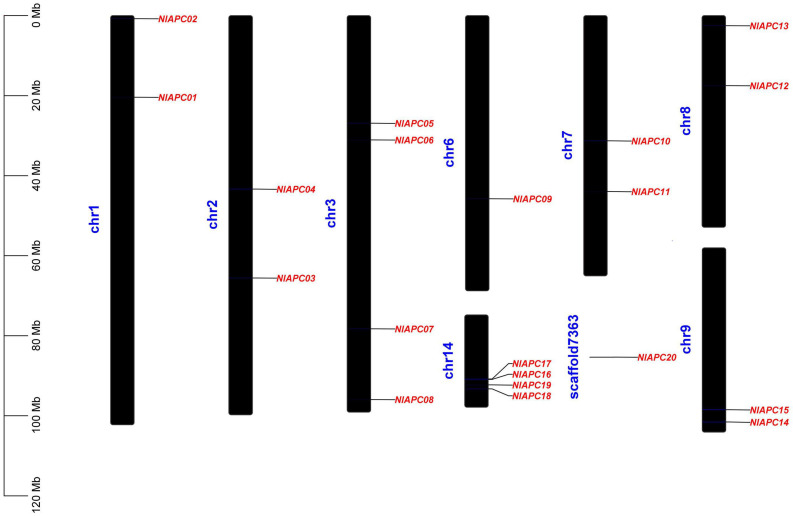
Chromosomal location of identified BPH APC transporter genes. The diagram was constructed by TBtools (version 1.089) software according to the gene annotation information (gff3 file) of BPH genome. Chromosome length is indicated by the scale bar on the left.

Based on the genomic location information of each APC gene family member, all 20 APC transporter genes were mapped onto the BPH chromosome (Figure 3). The results showed that BPH APC transporters were widely distributed on eight autosomes (chr1, chr2, chr3, chr6, chr7, chr8, chr9, and chr14) out of the total 16 chromosomes and an unassembled scaffold of number 7363. Statistical analysis indicated that there was no positive correlation between the length of a BPH chromosome and the number of APC transporter members (*P* = 0.715, Pearson’s test). In particular, four APC members (*NlAPC16*, *NlAPC17*, *NlAPC18*, and *NlAPC19*) were concentrated on chr14, whose length was the shortest in all BPH chromosomes.

### Phylogenetic Analysis of APC Genes Among Five Hemipteran Species

**FIGURE 4:**
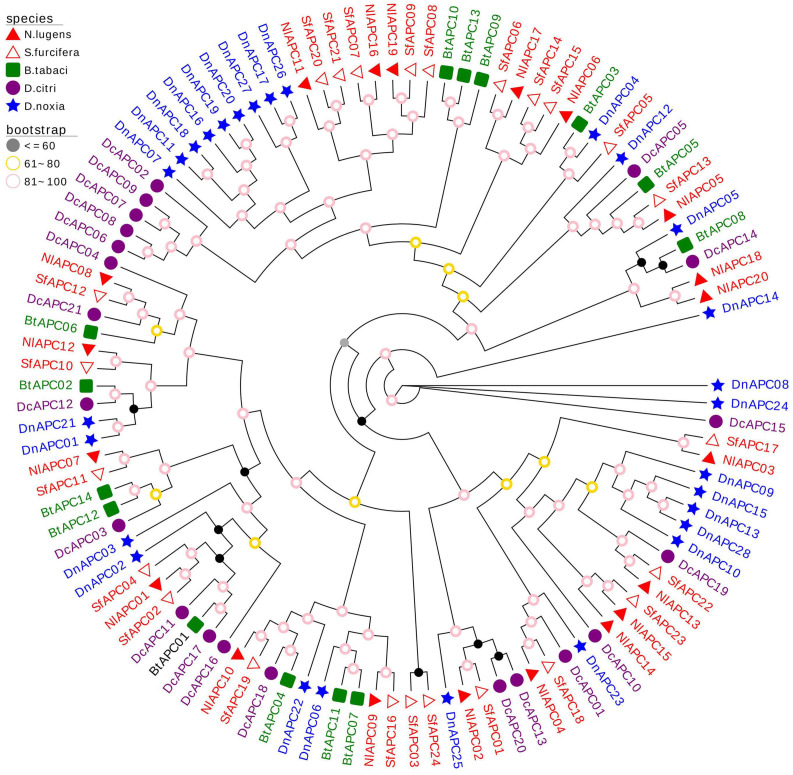
Molecular phylogenetic analysis of 107 APC transporters from five hemipteran species, including *Nilaparvata lugens* (20), *Sogatella furcifera* (21), *Bemisia tabaci* (14), *Diaphorina citri* (24), and *Diuraphis noxia* (28). The phylogenetic tree was constructed through the Maximum- likelihood (ML) method with 1,000 bootstrap replicates, using the best-fit model of VT + R10 in IQ-Tree software version 2.0. The APC genes from the same family (Delphacidae, Aleyrodidae, Psyllidae, and Aphididae) of insect were marked with the same color.

To explore the evolutionary patterns of the APC transporter family in hemipterans, the APC family members from the other three hemipteran insects were identified and phylogenetically analyzed, along with *B. tabaci* as reported by Xia et al. (2017). These insect species were specifically chosen to provide polyphagous (*S. furcifera*, *B. tabaci*, and *D. noxia*) and oligophagous groups (BPH and *D. citri*), as well as diverse insect taxa, including Delphacidae (BPH and *S. furcifera*), Aleyrodidae (*B. tabaci*), Psyllidae (*D. citri*), and Aphididae (*D. noxia*). The obtained results suggested that there was no significant difference in the number of identified APC members between the polyphagous (the number of APC members identified in *S. furcifera*, *B. tabaci*, and *D. noxia* was 24, 14, and 28, respectively) and oligophagous insect species (20 and 21 APC genes were identified in BPH and *S. furcifera*, respectively). Following this, an ML phylogenetic tree with 1,000 bootstrap replicates was produced based on the best-fit model of VT + R10 (Figure 4). The phylogenetic tree of multiple species displayed a clustering pattern of interfamily conservation and lineage-specific expansion of the APC gene family. For example, both belonging to the same family of Delphacidae, most BPH APC family genes clustered with those from *S. furcifera*. In contrast, some paralogs of APC genes from *B. tabaci*, *D. citri*, and *D. noxia* were assembled into several separate clades, demonstrating family specific gene expansion.

### Stage- and Tissue-Specific Expression Profiles of APC mRNAs in BPHs

**FIGURE 5:**
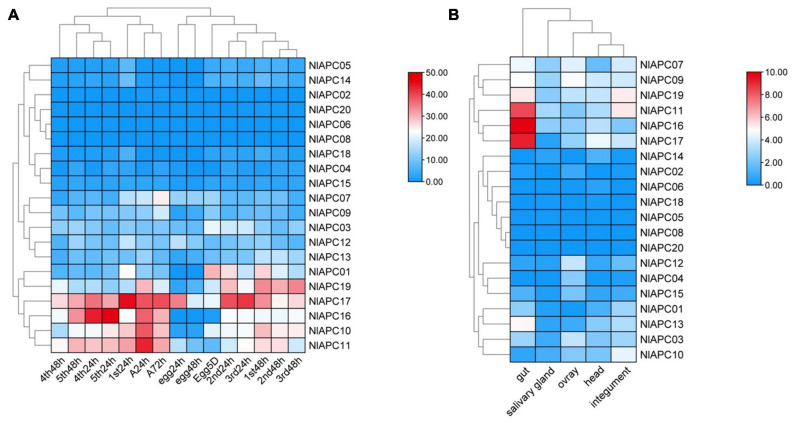
Spatiotemporal expression profiling of identified APC transporter genes in BPH based on large-scale transcriptome data. Kallisto v0.46.1 was used to calculate the TPM (the transcript abundance in transcripts per million) value for each identified APC gene base on the RNA-seq data downloaded from NCBI database. **(A)** Developmental stage-specific expression patterns of APC genes in BPH. **(B)** Tissue-specific expression patterns of APC transporter genes in BPH. Log_2_(TPM + 1) was used to scale the gene expression level of each APC transporter in different tissues.

Using the transcriptomic data, gene expression patterns of 20 BPH APC transporters were determined at 15 different developmental stages (egg-24 h, egg-48 h, egg-5 days, 1st-24 h, 1st-48 h, 2nd-24 h, 2nd-48 h, 3rd-24 h, 3rd-48 h, 4th-24 h, 4th-48 h, 5th-24 h, 5th-48 h, female adult-24 h, and female adult-72 h) (Supplementary Table 3) and in five different tissues (head, salivary glands, integument, gut, and ovaries) (Supplementary Table 4). Developmental expression analysis results showed that although most BPH APC transporters had relatively low expression levels throughout all of the tested developmental stages, several APC members (*NlAPC01*, *NlAPC10*, *NlAPC11*, *NlAPC16, NlAPC17*, and *NlAPC19*) had relatively high transcript abundance at certain developmental stages (Figure 5A). In particular, *NlAPC17* was highly expressed during almost all of the examined stages. Moreover, according to the clustering model of gene expression revealed by the heatmap, the growth stages of the BPHs could be divided into four groups, that is, eggs, younger nymphs (1st, 2nd, and 3rd nymph), older nymphs (4th and 5th nymphs), and adults, among which the stages of older nymphs and adults had a relatively higher expression than eggs and younger nymphs, except for 1st nymph 24 h, implicating the potential functions of APC amino transporters in the growth and reproduction of BPHs.

Similarly, significant expression fluctuations in different tissues were also observed in most BPH APC genes (Figure 5B). Among these 20 BPH APC transporters, the expression of *NlAPC08* and *NlAPC20* was not detected in any tested tissue, whereas *NlAPC05* was found to be specifically expressed in the salivary glands. In the remaining 17 APC members, 7 (41.2%) and 5 (29.4%) genes had their highest transcriptional levels in the tissues of the gut and ovaries, respectively. It is noteworthy that *NlAPC11*, *NlAPC16*, and *NlAPC17* showed extremely high levels of expression in the gut, with TPM values of 337.7, 892.8, and 587.8, respectively (Supplementary Table 4). The above results suggested that the tissues of the gut and ovaries might be the main regions where the APC transporter plays a dominant role.

### The Role of a Member of the APC Gene Family in Regulating Fecundity in BPHs

To screen the APC members that might be involved in the regulation of fecundity in BPHs, the temporal and spatial expression patterns of all BPH APC members were comprehensively evaluated, and the APC member of *NlAPC09* was selected, which reached its highest gene expression levels in both adult insects and ovaries compared with other growth stages and tissues (Figures 5A,B). Furthermore, the function of *NlAPC09* in modulating fecundity in BPHs was verified by qPCR and RNAi experiments.

**FIGURE 6:**
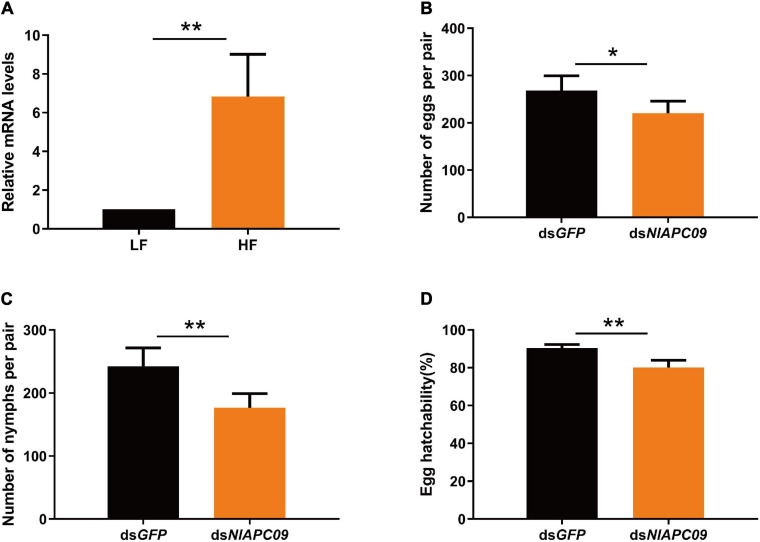
The determination on the role of *NlAPC09* in regulating fecundity in BPHs. **(A)** Relative expression levels of *NlAPC09* in the ovaries of BPHs from the high-fecundity and the low-fecundity population as evaluated by qPCR. HF, high-fecundity population; LF, low-fecundity population. The effect of silencing *NlAPC09* expression on the egg laying amount **(B)**, the nymph number **(C)**, and egg hatchability **(D)** in BPHs. Student’s *t*-test was used to compare the difference in the mRNA level of *NlAPC09* between different populations and in the fecundity parameters between different treatments (*P < 0.05; **P < 0.01).

The results of qPCR analysis showed that *NlAPC09* exhibited a significantly higher expression level (6.8 times) in the ovaries of the high-fecundity population than in the low-fecundity population (*P* = 0.005, *t*-test) (Figure 6A). RNAi results showed that the mRNA expression levels of *NlAPC09* were significantly decreased at 24 h (66.7%), 48 h (41.7%), and 72 h (34.7%) after administration of ds*NlAPC09* (all *P* < 0.05, *t*-test) (Supplementary Figure 3). Compared with the control group (injected with *GFP* dsRNA), the fecundity of the female insects was significantly lower in the ds*NlAPC09-injected* group, in which the amount of oviposition was reduced by 17.8% (*P* = 0.016, *t*-test) and the nymph number was reduced by 27.2% (*P* = 0.001, *t*-test) (Figures 6B,C). In addition, administration of ds*NlAPC09* significantly reduced the hatching rate of BPH eggs (*P* < 0.001, *t*-test) (Figure 6D). These results indicate that the APC transporter gene of *NlAPC09* might play an important role in regulating fecundity in BPHs.

## Discussion

In the current study, a total of 20 putative AAT genes belonging to the APC amino acid transporter family were identified in a notorious sap-feeding pest, BPH. Furthermore, their protein features, structural characterization, evolutionary patterns, and potential functions in reproduction regulation in this insect were analyzed.

### Functional Domain and Gene Structure Features of APC Transporters in BPHs

Molecular trait analysis showed that the APC family members classified into the same subgroup by phylogenetic tree displayed a similar conserved motif distribution (Figures 2A,B). Nonetheless, relatively high variabilities were present in in protein features, motif composition, and gene structure characterization among these putative APC transporters in BPHs. We deduced that the high variabilities among APC family members could be attributed to their substrate specificity, transport mechanism diversity, and significant functional divergence (Wolfersberger, 2000; Uchino et al., 2002; Boudko et al., 2015), which has been well-revealed in another type of AATs of the solute carrier family 7 (SLC7) in *A. aegypti*. For example, *AaCAT1* functions as a Na^+^-independent AA transporter with a unique selectivity to L-histidine, whereas its *AaCAT1* paralog of *AaCAT3* is a Na^+^-dependent transporter that transports essential cationic and neutral amino acids with a preference for arginine (Hansen et al., 2011; Boudko et al., 2015). Therefore, it is important to relate the structural properties to their potential biological function for BPH APC transporters in future studies.

A strong negative correlation has been reported between intron length and the corresponding gene expression in eukaryotes, with low-level expressed genes possessing larger intron length than that of highly expressed genes (Castillo-Davis et al., 2002; Eisenberg and Levanon, 2003; Nott et al., 2003). Some previous studies also revealed that the presence of an excessive number of introns was unfavorable for the highly efficient expression of the corresponding gene (Jeffares et al., 2008). Our investigation of exon/intron structure in verified BPH APC genes revealed that a strikingly large number and sequence length of introns were contained by the majority of BPH APC transporter members, and this phenomenon was also found in other types of insect AATs (Ito et al., 2008). Therefore, most APC genes were expected to have relatively low expression levels in BPHs, and our *in silico* prediction was further supported by the spatial-temporal expression profiling of this gene family calculated from RNA-seq data (Figures 5A,B). From another perspective, the existence of a conspicuously large number of introns in the BPH APC gene family could also provide ample genetic resources for RNA editing and alternative splicing in post-transcriptional regulation, promoting the adaptive and evolutionary potential of this species without increasing the number of genes (Koralewski and Krutovsky, 2011; Ge and Porse, 2014).

### Evolutionary Pattern of the APC Transporter Family Among Hemipteran Species

To explore the evolutionary pattern of the APC gene family in hemipterans, APC family members from five hemipteran species were identified and subjected to evolutionary analysis. The results suggested a high variability in the size of the APC family among the examined insect species, ranging from 14 in *B. tabaci* to 28 in *D. noxia*, which might be owing to a series of complex factors involved in dietary nutrition, microbial symbionts, and evolutionary processes in the surveyed insect taxa (Duncan et al., 2016; Xia et al., 2017). Furthermore, our phylogenetic analysis demonstrated a significant family specific expansion of APC family genes in hemipterans (Figure 4), and this phenomenon has been well-reported and explored in many previous studies (Boudko, 2012; Duncan et al., 2014; Dahan et al., 2015). Price et al. (2011) and Dahan et al. (2015) considered that the interactions between obligate endosymbionts and insect hosts might lead to expansion of the AAT gene family in sap-feeding insects, whereas Duncan et al. (2016) proposed that the paralog evolution in insect APC family expansion was likely driven by many complex factors and processes, including both symbiotic and non-symbiotic traits.

Other than conspicuous gene expansion, a phylogenetic pattern was also observed in insect APC family genes during the evolutionary analysis, in which the vast majority of APC members from BPH clustered with those from *S. furcifera*, suggesting that the evolution of APC paralog precedes the divergence of the Delphacidae lineage. Our results on this evolutionary pattern of the insect APC gene family were consistent with those reported by Xia et al. (2017).

### Spatial-Temporal Expression Patterns of APC Genes Provides Insight Into Their Functions

Spatiotemporal expression profiling is commonly considered a valuable clue to infer the potential biological function of an uncertain gene (Das et al., 2016). Our investigation on the developmental expression of BPH APC genes showed that despite low-level expressions for most APC members, several BPH APC genes showed relatively higher expression levels in specific developmental stages, including older nymphs and adults (Figure 5A). From these results, we deduced that nymphs and adults are the two main feeding stages for the BPH species, during which large amounts of amino acids need to be transported between BPH and rice plants (Chen et al., 2011) or/and between BPH and its endosymbionts (Sasaki et al., 1996; Wan et al., 2014), thus it is reasonable that most APC members reach their highest expression levels during these two stages of the BPH life cycle. Similarly, Xia et al. (2017) also found that most APC transporter genes were highly expressed at the 3rd-instar nymph stage in *B. tabaci*.

Tissue-specific expression patterns of APC genes were determined in the head, salivary glands, integument, gut, and ovaries of BPHs. Following these determinations, we observed that the gut tissue possessed the highest number of highly expressed APC genes, followed by the ovaries (Figure 5B). Based on this, it could be concluded that the gut and ovaries were the two primary sites where BPH APC transporters were dominantly functional compared with the other three tissues. For this reason, we speculated that essential amino acids were primarily absorbed by the midgut in insects (Fu et al., 2015); therefore, a vast majority of the AA transporters were expected to be distributed in this tissue, as well as a large number of mycetocytes, which eventually led to high APC gene expression levels in the gut of the insects (Douglas, 1996; Wolfersberger, 2000; Stefanini, 2018). As for the APC genes that were highly expressed in the ovaries, it was postulated that they might participate in the regulation of reproduction, a function that has been reported for some other insect AATs (Evans et al., 2009; Hansen et al., 2011; Boudko, 2012). Therefore, the regulation of fecundity in BPHs was further explored using a single selected APC transporter in the current study.

### Effect of NlAPC09 in Regulating Reproduction in BPHs

Apart from amino acid transportation, AATs are also responsible for other life activities in insects, including nutrient signal transduction (Boudko, 2012), growth maintenance (Fu et al., 2015), fecundity regulation (Attardo et al., 2006; Boudko, 2012), neurotransmitter synthesis (Meleshkevitch et al., 2009), and viral resistance (Ito et al., 2008). Among these, the function of regulating reproduction by APC transporters has been of particular focus in recent years and is anticipated to be used in pest control in the future. Attardo et al. (2006) and Boudko (2012) reported that the inhibition of AAT expression through the RNAi approach significantly decreased vitellogenin gene expression and oviposition amounts in *A. aegypti*. Furthermore, they proposed that fecundity regulation in mosquitos by AATs might be associated with the AA/TOR signaling pathway. To sum up, two mechanisms might be involved underlying AATs regulate insect fecundity. On the one hand, AATs highly expressed in insect fat body tissue could directly take up AAs from the hemolymph and convert them into yolk proteins (Carpenter et al., 2012); on the other hand, as the nutritional sensors, AATs are also able to interact with the insulin/TOR signaling cascades and control the biosynthesis of the juvenile hormone (JH) and ecdysone. By modifying the concentrations of these endocrine hormones, AATs could indirectly affect vitellogenesis and egg maturation in insects (Smykal and Raikhel, 2015).

In this study, we identified a potential fecundity regulatory gene of *NlAPC09* to regulate fecundity in BPHs, which exhibited a unique pattern of adult- and ovary-specific high expressions. In addition, subsequent qPCR and RNAi experiments further confirmed the important role of *NlAPC09* in regulating fecundity in BPHs. It is worth noting that besides the decrease in the total number of eggs deposited and nymphs hatched (Figures 6B,C), RNAi-mediated knockdown of *NlAPC09* resulted in a significant drop in the hatching rate of eggs laid (Figure 6D), indicating that expressive suppression of *NlAPC09* had a remarkable impact on both oviposition quantity and embryo quality. The deficiency of essential amino acids significantly lowering the egg hatching rate has been reported in other studies (Hosokawa et al., 2007; Fuchs et al., 2014); however, the related mechanism has not been well-explored to date. Furthermore, whether *NlAPC09* regulates BPH reproduction through the AA/TOR pathway and the type of amino acids it transports remains to be elucidated in future studies.

## Conclusion

In summary, 20 putative APC transporters were identified in the BPH genome. Molecular trait analysis revealed that BPH APC transporter family members exhibited high levels of variation in protein features, conserved motif distribution patterns, and exon/intron organization. Phylogenetic analysis of APC genes from five hemipterans displayed an evolutionary pattern of interfamily conservation and lineage-specific expansion. In addition, spatial-temporal expression analysis revealed diverse gene expression patterns in the 20 BPH APC transporter members. Lastly, a potential BPH fecundity regulatory gene of *NlAPC09* was identified and was shown to participate in the regulation of fecundity in the insects through qPCR and RNAi-mediated knockdown technologies. Our results not only provide a foundation for further research on the molecular structure, evolution pattern, and biological functions of APC transporters in BPHs, but also offer new ways to control this pest.

## Data Availability Statement

The original contributions presented in the study are included in the article/Supplementary Material, further inquiries can be directed to the corresponding author/s.

## Author Contributions

LY, RP, and KL conceived and designed the study and wrote the manuscript. ZG, MZ, and LZ contributed to the materials. LY and KL performed the experiments. LY and RP performed the data analysis. All authors contributed to the article and approved the submitted version.

## Conflict of Interest

The authors declare that the research was conducted in the absence of any commercial or financial relationships that could be construed as a potential conflict of interest.

## Footnotes

1 http://www.insect-genome.com

2 https://www.ncbi.nlm.nih.gov/

3 http://pfam.xfam.org

4 https://web.expasy.org/protparam/

5 https://swissmodel.expasy.org/

6 http://www.cbs.dtu.dk/services/TMHMM/

7 https://www.genome.jp/tools-bin/clustalw

8 https://espript.ibcp.fr/ESPript/cgi-bin/ESPript.cgi

9 https://www.evolgenius.info/evolview/#login

10 https://meme-suite.org/meme/

11 https://www.ncbi.nlm.nih.gov/sra

## Supplementary Material

Supplementary Figure 1 The protein domains contained by 20 putative APC transporter identified in BPH genome. The identification and annotation of protein domains of BPH APC transporters was conducted through a web resource of SMART (https://smart.embl.de). Boxes of different colors represent the different domains, and the three types of domains, including AA_permease, AA_permease_2, and AA_permease_C, were considered as the featured domains of APC transporter gene family.

Supplementary Figure 2 The sequence logo of 10 conserved motifs predicted in BPH APC transporter proteins. MEME Suite 5.5.3 was used to analyze the conservation of amino acid residues contained in the predicted conserved motifs of BPH APC transporter proteins.

Supplementary Figure 3 The expression level of *NlAPC09* in the ovaries of BPHs at 24, 48, and 72 h post-RNAi. β*-actin* gene was used to normalize the gene expression of *NlAPC09* in BPH. The mRNA level of *NlAPC09* in the ds*GFP*-treated BPHs was set to 1. Single asterisks (*P* < 0.05) and double asterisks (*P* < 0.01) indicate significant differences in mRNA level of *NlAPC09* between ds*GFP*- and ds*NlAPC09*-injected groups.

Supplementary Table 1 The primers used in this study.

Supplementary Table 2 The amino acid sequence of APC transporter genes identified in four hemipteran insects.

Supplementary Table 3 TPM values of APC transporter genes of BPH at different developmental stages. Stage-specific gene expression level of APC transporters was calculated using Kallisto v0.46.1 based on a series of RNA-seq data downloaded from NCBI database. A total of 15 different developmental stages were evaluated in this study.

Supplementary Table 4 TPM values of APC transporter genes of BPH at different tissues. Tissue-specific gene expression level of APC transporters was calculated using Kallisto v0.46.1 based on transcriptome data obtained from NCBI database. A total of 6 different tissues were evaluated in this study.
