# Supplementary figures and images for: Genome-Wide Identification and Characterization of Amino Acid Polyamine Organocation Transporter Family Genes Reveal Their Role in Fecundity Regulation in a Brown Planthopper Species (Nilaparvata lugens)
Source: Front Physiol. 2021 Jul 14;12:708639. doi: 10.3389/fphys.2021.708639 (PMC8316623; doi:10.3389/fphys.2021.708639)

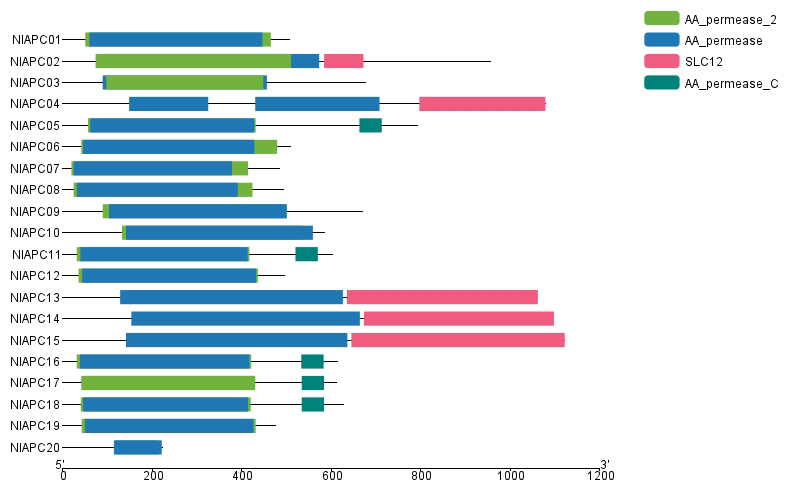

Supplement: Supplementary Figure 1 — The protein domains contained by 20 putative APC transporter identified in BPH genome. The identification and annotation of protein domains of BPH APC transporters was conducted through a web resource of SMART (https://smart.embl.de). Boxes of different colors represent the different domains, and the three types of domains, including AA_permease, AA_permease_2, and AA_permease_C, were considered as the featured domains of APC transporter gene family. [file Image_1.JPEG]

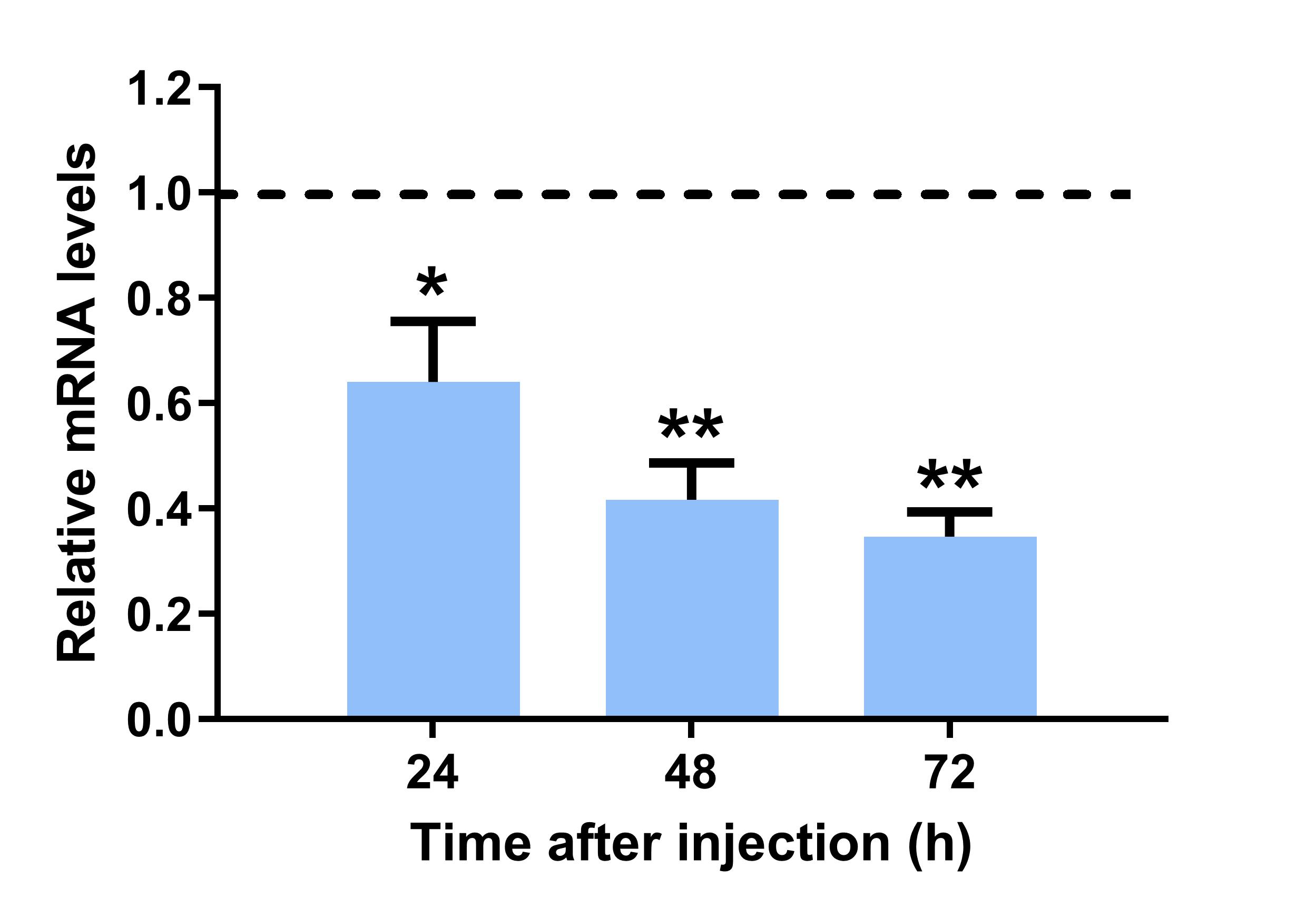

Supplement: Supplementary Figure 3 — The expression level of NlAPC09 in the ovaries of BPHs at 24, 48, and 72 h post-RNAi. β-actin gene was used to normalize the gene expression of NlAPC09 in BPH. The mRNA level of NlAPC09 in the dsGFP-treated BPHs was set to 1. Single asterisks (P < 0.05) and double asterisks (P < 0.01) indicate significant differences in mRNA level of NlAPC09 between dsGFP- and dsNlAPC09-injected groups. [file Image_3.JPEG]
