# Supplementary material for: Genome-Wide Identification and Characterization of Amino Acid Polyamine Organocation Transporter Family Genes Reveal Their Role in Fecundity Regulation in a Brown Planthopper Species (Nilaparvata lugens)
Source: Front Physiol. 2021 Jul 14;12:708639. doi: 10.3389/fphys.2021.708639 (PMC8316623; doi:10.3389/fphys.2021.708639)
Supplement: Supplementary Table 1 — The primers used in this study. [file Table_1.DOCX]

Supplementary Table 1. The primers used in this study.

| Primer | Primer sequence (5'-3') | Purpose |
| --- | --- | --- |
| *NlAPC09-F* | GTGTAATGATAGGTGCTCTCTCGT | Quatitative real-time PCR |
| *NlAPC09-R* | CAACCACCCAGGCATAGAGG | Quatitative real-time PCR |
| *dsNlAPC09-F* | AGTGTAATGATAGGTGCTCTCTCG | synthesis of dsRNA for *NlAPC09* |
| *dsNlAPC09-R* | GCCCACTGTAGAGTGCCAAT | synthesis of dsRNA for *NlAPC09* |
| T7- *dsNlAPC09-F* | GGATCCTAATACGACTCACTATAGG AGTGTAATGATAGGTGCTCTCTCG | synthesis of dsRNA for *NlAPC09* |
| T7- *dsNlAPC09-R* | GGATCCTAATACGACTCACTATAGG GCCCACTGTAGAGTGCCAAT | synthesis of dsRNA for *NlAPC09* |
| *dsGFP-F* | AAGGGCGAGGAGCTGTTCACCG | synthesis of dsRNA for *GFP* |
| *dsGFP-R* | CAGCAGGACCATGTGATCGCGC | synthesis of dsRNA for *GFP* |
| T7- *dsGFP-F* | GGATCCTAATACGACTCACTATAGG AAGGGCGAGGAGCTGTTCACCG | synthesis of dsRNA for *GFP* |
| T7- *dsGFP-R* | GGATCCTAATACGACTCACTATAGG CAGCAGGACCATGTGATCGCGC | synthesis of dsRNA for *GFP* |

Supplementary Table 2. The amino acid sequence of APC transporter genes identified in four *Hemiptera* insects.

| Gene | Amino acid sequences |
| --- | --- |
| *NlAPC01*  (*Nlug07038-TA*) | MTITSRSVSAEKLRSPEEVPLQPSAAQSSGNVADTAGDGDDGVCLKAKMSLLNGITVIVGSIIGSGIFVSPTGVLVSTGSVNMALIVWTASGIFSMVGAYCYAELGCMISKSGADYAYIMETFGPFLAFMRLWVECMIVRPCSQAIVALTFSIYVLKPFFPDCQPPEESLRLLAVCCILILTFVNCWDVKWATRVQDTFTYAKLLALCIIIGAGVMKLIEGKTQYFTFENTTYEPTSIALSFYSGLFAYNGWNYLNFIIEELKDPIKNLPRAIAISCTLVTIVYVMTNVAFYTTLSPKEVLQSEAVAVTFANEIFGYLAWTIPVFVAMSTFGAVNGILLTSSRLFYAGACEGQMPEILTMIQIKRLTPTPAVLCIALLSLLYLTVSDIQALINYVGFATWLSIGVSVLCLPVLRYTQPALKRPIKVNLIFPIVYIVATILVTVVPMIAEPKQTGIGCLMIFTSVPVYLVFIAWKSKPIFFQKAVVTATRFLQKTMLVVGKQNPAKV |
| *NlAPC02*  (*Nlug21226-TA*) | MTDPSAFSINSGHEAPRINPENENIPLISRNSFFRKITKLCGGRDMDSGTAENGYVEFGSIGEISNGRTLGTFAGVFSPVALSMFSALIFLRVGYIIGNAGLITTLIQFAIAYGILFFTVASICAISTNGAVEGGGAYFMISRTLGPEFGGSIGTLFFLANVVSSALYIVGFGEGLIENFGKTGYLMNEDSKFYLPSGEWWQFLYCTVLNVVNLLVCLIGAGMFAKTSVAILGVVTVSLISVVVSLSVQSSFEVAIPERNSLFTNSTYHANGTFTGLSTATLRNNLYPSYAPDYTSTSELTNFASVFGVLFSGVTGIMAGANMSGELRDPAKNIPKGTLSAVAFTLCCYLTISVLIAATCDRFLLQNNYIFMLPTNVWPPFVTIGILTATFSASLSNLIGSSRVLEALAKDNVFGQLLMFISKGVWRSNPLAAVFLSWLLVQLILLIGSLNLIAQINSVLFLLSYLATNLACLGLDLASAPNFRPSFKYFSWYTALIGLIGTLVMMFVINSVYAASSIVLCLLLVLLLHLFSPSRSAHWGSISQALIFHQVRKYLLMLDSRKDHVKFWRPQMLLMVGNPRSCCPLISFVNDLKKSGLYVLGHVKVGPEFSQMSDDPTLDEYSHWLSLVDHIKVKAFIELTVARSVREGLHHLVHLSGLGAMKPNTIVFGFYDDQIPHDYFLDADSPYRTERFQQECGGASGGGSEGESGRGSGGGSEVFGLRQSVEEKQLSGQEFVCLISDVLRMKKNVCVCRHFHTLNKAKAAVKSGSFKYIDVWPVNFFHPNAEDPFNTTSLFMLQLACIINMVRGWKHLQLRVFLCDSNMAVTGLTEFHAQRSTEQRFRHLLHSLRIKASIHPVAEWSTQLASLRGGSVFRTKDHEVSDGHVAKTYLSNLNQVMRHHSSDKATALMLLYLPEPPSAQSSANDLTQYLQYLNQLTTDLPATLLVHGISTVTSTTL |
| *NlAPC03*  (*Nlug07271-TA*) | MEPQLASSLNGRQKKTNEVDWSKYGLRTEQQDSSALSWKNSPDEGGYGAGGHQPADVSELFAGEQNNEPWWKSQFFISQPVLFGVWDGVFTSCLINVFGVIVFLRSGWIVAEAGVANAVLMVLATVGIALISVLSAVGICERCRMESGGVYFLLAHVLGSRIAAAVGLLYVFGQAVGCALFVLGFGESVAGLVGLGHSVWAQRGVGSAAILLLGVINVAGVKWVVKLQFLLLLVLLLAGLDFAVGSFIKTDIKNGFDGWLTGKLSENALPAYSAGNNWFTVFGVFFPTITGIMAGINMSGDLRQPTTDIPNGTLLAVATTTGLYLMFVLVLGSTCTRVALRTDYMIAAKVSALHVMLLAGLYVSSMSSCLGAMYGTPRVLQSIANENVIPIILLTFSLPIPYYRGPNRVPVYSMGVVALVTMGFLLVGDINSLAPIVTMPFLMMYAALDYAYFALAQTFDLLHHREQRFRSTQSGRSYGSSPSLQHSDISNDLDSLFPERSHHRTYTSITSESSPTIEQQPQPQQQNGASSRMVHSKRGYWYSSWCNRWLSLLGALLKIGMMFLVHWSYALANCGVVLVIWIYIGLANPAVKPGVAIHFRLLRWLHAVLLRLCGKRTVEFEEIVVPPDTPGVHLSPSQLTDENEDFANRQRFHQSATIRPIPDSEHAARYGDVQILR |
| *NlAPC04*  (*Nlug07490-TA*) | MSSSINMERFKVTRSDDKKAKYGATAAAGSNAEVETSLMRANLDMDFNQDSIGEYTVTNPQLIPAGGSGPTRRAIRDGTDDQPFVSEKEKMSGYETNLYLYSEELEDRPRISTLLGSLANYENTIPSTPADPDAKPSAGGARMGTLVGVYLPCIQNIFGVILFIRLTWVVGTAGAVQGFLIVLTCCCVTMLTAISMSAIATNGVVPAGGSYFMISRSLGPECGGAVGMLFYTGTTLAAAMYIIGAVEIVLTYMSPSLSIFGDFTKDANIMYNNFRVYGTGLLMVMGTIVFVGVKFVNKFASVALACVILSIVAVYVGIFVNFNGNDKLKMCVLGNRLLKDIHIDQCNKTVGGELYNLFCPGNSTKSCDPYFLANNVSIENGIKGLASGVFLTNLGDSFLEDGQYIAKSLDAEEINRLDRPTYNQVMADITTSFTLLIGIFFPSVTGIMAGSNRSGDLADAQKSIPIGTICAILTTSTVYLSAVLLFAGTVDNLLLRDKFGQSIGGRLVVANIAWPNEWVILIGSFLSTLGAGLQSLTGAPRLLQAIAKDGIIPFLAPFAVSSSRGEPTRALLLTITICQCGILLGNVDYLAPLLSMFFLMCYGFVNLACALQTLLRTPNWRPRFKYYHWSLSFIGLALCIAVMFMSSWYYALVAMGMAGLIYKYIEYRGAEKEWGDGIRGLALSAARYSLLRLEEAEPHTKNWRPQILLLAKLTSELVPKYRKMFTFTSQLKAGKIQTTGLGGMKPNTVILGWPYGWRQSEEEQSWHTFLHAVRVITASRMALLVPKGINFFPDSTQKMSGNIDIWWIVHDGGLLMLLPFLLKQHRTWKNCRLRIFTVAQMEDNSIQMKKDLKTFLYHLRIEAEVEVVEMTDNDISAYTYERTLMMEQRNKMVRELRLNKKESLGMVDFNEIPSEENMPLTVDSATNTEEVQAIVDHHHDIKATKNQEQKSRTIRFQEPSEKDEDKQEQNQTDGSSEKSPTPSNKSPAAANNVSSGDNPAVTPDENNVRRMHTAVKLNEVISKRSYEAQLVILNLPGPPRDTKAERESNYMEFLEVLTEGLERVLMVRGSGREVITIYS |
| *NlAPC05*  (*Nlug01129-TA*) | MKIPTPGLFPTGDYRKDGLALFTKLIRTKNVEGMQGELPPRLHAKSDKKLKLKKCLTTLDLTSLGVGSCVGTGMYLVAGMVAKNVAGPGVVISFIIAAIASIFSGACYAEFGVRVPHTTGSAYMYSYVTVGEFVAFVIGWNMVLEYLIGTSACACALSACFDALTDGAISSLVTESVGTFFGRPPDFLAFVITLLMMLLMAAGVKKSLVFNNILNAINLSAWVFVMTAGMFYVNTANWSEHKGFLPYGWSGVFTGAATCFYAFIGFDIIATTGEEANNPKKSIPLAIVTSLIIILIAYVTSSMMLTLIVPYEKVDQDSALVEMFGQVGAWRCKLVVAVGALAGLSVSMFGSMFPMPRIVYAMAQDGLIFRTLAQVFPLTGTPVVATFGSGFAAAIAALLISLEVLVEMMSIGTLLAYTLVSTCVLILRYQPHSTNLVDLLPESLRTPVKGSPSKETLSNGQVSYGNQLHPDQLRSALGGVPAQQPPAGPQPSPGLTSTLTQQQQQQQRIMVRRVTRSSPDSDDTFPGEEPDEFSMRDDQFLVADRSENKFYGTVHGGSSAGGGGVAGPTLGAIGRRLQAVTYLCPAIFPWVDSGPATEDSGMFVMKMVGILYILILIFDLIIVLGMGSDSSIVTFLVIFFLLCIIALLLIISRKPQNRKTLMFMTPGIPFVPAIAVIVNIYLIFKLSILTLVRFTVWMIIGLLVYFYYGIKHSSLEEEEDPQNIELSVTTEKPPPAQQTVDSERETPAWDSASYHQPQQASSPYTSIQQPPSSGSNRAPLFVSPQQFPTWDD |
| *NlAPC06*  (*Nlug02200-TA*) | MYSALTMNQKVYRSRIWKKFVRKKAVDRARMQQTELRRALDLIDLTTFGIANTFGLGVYVLAGMGARVAGPGICVSHFIVGVATTITALCYGELAAAVPRSGSVYSYTYVLMGEFMAFIFGWLQCLENIIGVACLGGGLSTYIDNMIDNKLADYFAEINIPVVLAEPNVFAAFLIAICSCILVGGVELSVTFQKLLTFLNLTTLTIIIVIGVCNADLKNWSWKPGDEDFPTDGSGGSGGFFPFGFHGVLVITRHCVYAYRGFEGISCAGEEAKNPRKLIPLAILLTLIIASVSYIGVAVALTLMWPYFDESITSPFPYALDMTNMHNVKWVVSTGILFCLCACLLGNTYTCTRVLYSMGADGLILKWFGKVNSKTKTPIFSTVATALLSGAFAIILGTDDFVRTIVSIVNLVSHTVVSVCVLLLRYRNVHEQCSQHEQDGSPIRMEVLLNLKAHSKTPTAQSYLIVKITILIMCLLIYFSYGIGHSVENTEKEQPSESGSCFQSNSASMT |
| *NlAPC07*  (*Nlug06371-TA*) | MADDANRVTLRRKITLANGVALIVGSIVGSGIFVSPAGVFLYTRSVAASLVVWTLSGAFSAVGAVCFAELGTCIARSGGDYAYILEAFGDLPAFLYLWVVLLVIRPCTQAVVALTFAQYAAKPFFPTCEPPPVAVSLLAAACLCLLTAVNCVSVRWSMRVQNVFTWGKLIALVAIIVAGVYHIFTGGTSNFENAFEGKYDVGSIALAFYSGLFAFGGWNFLNFVTEELQDPYKNLPRAIWIAMPIVTLIYVLANLAYFAVVSADEMLSSAAVAVTFGNKMFGALSWTVPVFVALSTFGGVNGILFTSSRLFLTGARQGHLPQVLAYVHVTRCTPVPSLVVTCVVSLLMVTNSNVFTLINYFSLVLWLSIGACIGALLWLRVSQPHLHRPITVHLALPVSFLLCCVFLIAVSAVTEPLNAVIGLLVIVAGIPVYYLCVKTRNEGKSIRKFNESMTVFIQKLFNVVSLDDDDDNHREDNDDDEKVIK |
| *NlAPC08*  (*Nlug19179-TA*) | MSTTKADSSSNSGATGGVQLQKKLGLLDGVAIIVGIIVGSGIFVSPKGVLIRSGSVGLGLVIWVLSGVLSVVGALCYAELGTMIPKSGGDYAYINEAFGALPAFLYLWVALLVIVPTGNAITALTFAEYIVKPAWPHCDPPYEVTRLIATLITCLLTAVNCYDVKWVTRVQDFFTATKVLALLMIVGVGVWALLVDSHTQNISQPFEGSNTSPGAIALAFYSGLFSYAGWNYLNFVTEELRDPYKNLPRAISISIPLVTVIYCLVNLAYFVILPKVDLLSSNAVAVTFADRALGGFAWSMPTFVACSTFGALNGAIFASSRLFFVGARQGHLPKAIALINVDTCTPVPSLIFLCIVTVVLLFIEDIYMLINYVSFVEALFTLISVSGLLWLRYKMPDRHRPIKVNILLPIAFFVICVFLVTLPVLERPQEVCVGLIITLSGIPVYYIFIEWNKKPQWIQKTSNYLNSTCAKMFMCVLEEGMVYKPMLEKKQEND |
| *NlAPC09*  (*Nlug06688-TA*) | MSSRYSPMDLIHRRPHSKGCEKDHMLEKINLCLTNESEKKECKPDEDENEKEEEEEHFIPSKQSSLLNGRPIPESDVEPTRNVQLQRELGLFSAVSLILSVMIGSSVGLTTFISQPGTTAVLLIFEFSKCIMICFAGALSFSELSTVVPRSGAEYSYFRVAFSKLHPFLGPLPCFLYAWVVVLILRPAEIAIIILTFTEYVYSPVVTLSGFKITPPYQMLLKKLLSLLTLGTISYINFSSVKLFVKLQNIFSSFKIVACFVVILGGVYSLSAGNVSNLNTGFEGSKTSLRDVVLALYSGLWAYDGWSSVTVVAEEIKNPEKNIFRSILIGVPLVTILYFFMNVSYMSVLTIPEMTAAPAVAEAFGEKVMGSLSIIIPIGVALSTFSCSLSVQFGVSRLCYAAGREGHMIEAFSYIHVRKCTPAPAVALQGLLTTIFILAGDISTLIEFASFLIWIFYGLTMVALIVLRYKKPLANRPYKVPIIIPISLAILSVILASVPIVFNPQIQYICALIFIALGLCVYYPFVYLKYRLPFMDKFTYLIQVMLEVVPPPSKPDDTTPSQTTMTPQPSVITANGSAANVSATANSSAANVSATANGSAANVSATANGNLANGAAFHGLTPMPISVHSISSTNGMATALPANGVIVTSSSNAIIQPPVASQQSLIVDHYT |
| *NlAPC10*  (*Nlug07560-TA*) | MSEESTAPIRAAVKHWLSASTRCRRSQLSPLSHSQCIPGHEPKSTRYTRSAHCEMHHDGRHIVHRSNVQAATAAAAVGRGPGGTGESDANGGLWGAGGQDDSSEMGGAAGGDQLERADATQDDALHLQRRVGLFSGVALIVGTMIGSGIFVSPSGLLIRTRSVGMSFLIWIACGVLSLLGALAYAELGTMNTSCGAEYAYFMDAFGSLPAFLFSWVSTLVLKPSQLAIICLSFAKYVVEAFVTECEPPETVVKMVAILSIMVILFINCYSVNLATGVQNAFTAAKLVAILIVICGGGYKLFQGNTQNFENMFSGETASIGSIATAFYTGLWAYDGWNNLNYVTEEIKKPSKNLPRSIMIAIPLVTLCYVLINISYLSVMSAAEMEESEAVAVTFGNRILGALAWLMPLSVTISTFGSANGTLFAAGRLCFAASREGHLMNILSYVHIRRLTPAPGLIFHSIIAAAMILSGNIESLIDFFSFTAWIFYGGAMLALIVMRYTRPNFPRPYKVPFIIPVVVLVISIYLIIAPIIDSPQVEYLYAALFILAGLIVYVPIVHYRFNTSIMDRFTVFCQLLLEVAPTQALFD |
| *NlAPC11*  (*Nlug13938-TA*) | MRSAHLIRVLTRRKTDDRCEDAGTPKLARVLGLFDLCALGVGSTLGVGVYVLAGAVARHDSGPAVTLSFLVAAIASAFAGVCYAEFAARVPKAGSAYVYSYVSVGELIAFIIGWNLVLEYIIGTASVARGFSNYVDSIMGNVMRDTLTEYLPLNMSFLSPYPDFLSCTVVLVLSLLLAWGVKESTLMNNIFTIVNLITVVTVIVCGSMKADLKNWSLHDVPAKAGSGGFMPFGISGVMAGAAKCFYGFIGFDCVATTAEEAKNPQRNIPLSIIFSLIIIFLSYFGIATVLTMMYPYYLQDPYAPLPYAFEKVGMPAVKVMVTVGAIFALCASLLGSLFPLPRMIYAMAIDGLLFRKFALIHPKWLTPINATLFAGFIGAIMAALFNLEQLIDMMSIGTLLAYTIVAMCILVLRYRDVNPTGSFEHNRKVKSNVAMDKFVTLFNLTSSKHPSSASEQIANWSIALLIASLAAVCLCLIHLEGQIVAGSYWAISLLSILSAISLIVLLVIYRQPQNDTVLSFKAPGIPLVPALSIFMNTFLMLKLDMHTWIRFSIWLFIGMIIYIFYSIPNSVEGLKDRLYDNHVKLDIRSKSDNKYASQTTKL |
| *NlAPC12*  (*Nlug00063-TA*) | MTKQVTSPVSDTCLMTPTSDTAPIGDDKVRMKKQLGLLEGVAIILGIIFGSGIFISPKGVIQEVGSVGLSLVVWAMCGVLSMIGALCYAELGTSIPKSGGDYAYIYEAFGPLFAFLYLWDAMLIFVPTTNAIMGLTFAKYVIDPFFPECPLPEVSVRFIAACAICFFTFMNCYNVKLTSNIQNSFMFGKTGALALIIIVGIASFFMNDVNNFDAPFQRSTFSPGPIAVAFYSGIFSYSGWNYLNFMTEELKNPYVNLPRAIYISLPLVTLTYVLANVAYLAVLSPDEMIASNAIAVSFGYRTLGVLSWIMPLMVAMSALGGLSVHIMTSSRMCFVGARYGHFPAMLSHLNINKLTPMPSLVFLNILSLLMLCTSDIHLLITYASFVESFFLLLSVSGLLWLRYKKPNMHRPIKVSLIVPVTFVILCIFLVIFPVFSAPYEVLMGVLITLTGIPAYYFGVVWKEKPSWFTNALDGTTAFVQKLFMSAKEEGVEGFD |
| *NlAPC13*  (*Nlug11217-TA*) | MSGRFNVQKVGHDNAGLTLDEGVIHTGRAGLSPDAVSSDNLPTGNDRKTSFTHYTLEALPRPEYYKSSGSGVKRPSLGELCGEEVVFSKETYKDPIKPNVHNVEASGPEDQTDEQEHTGGIKLGWINGVLVPCLLNIWGVMLFLRLSWVVAQSGISGTLIIIAISSVVCIITTLSLSAICTNGEVKGGGIYYIISRSLGPEFGASVGVIFASANAVSVSMNTIGFCNSLNDLLAEHGLQIIDGGDNDVRIIGVITICIMTVICAVGMEWESKAQNFLIVVIIAAIFDFLIGACLGPSSDLDRVKGFTGLSTEVFQENWGANYRKSEGDEQNFFTVFAIFFPSVTGIQAGANISGDLKDPSSAIPKGTMLALLISMVSYVLFVLFAGAAALRDASGMVTEMLPNATLPYWNCTSRVCDYGLHNSYTVMQLMSSNGNLIYAGCFAATLSTALTNLLSVPRLIQALGIDRIYPGLIFFSKGYGPHKEPYRGYVLTFVISTLFLMIAHLNVIAPLISNFYLASYALINFCTFHAGLVRPLGWRPTFRYYNTWLSLLGSFMCVVIMFLINWITSLLTFVIFFALYLVVVYRNPQVNWGSSTQAQTYKTALMTAYKLSNSTEHVKNYQPQLLVLSGQPQSRPPLIDLASLITRNNSLCICAEICKNRLNYKLRSVRTHKGMSWLGVRKIKSFFVVVDGVRMFDAGARALMQTSGVGRMRPNVLMMGYKNNWQSCPSEEMFSYFNTLHDAFENRLAVIILKMADGLDSSPFSGENDLDLDSGSTLDLTVGGGDVISHTGSLLHNDSSTSLNMPLQQNVLSQTSLTISGSKTNNNSARPIVIPTNAVSNHSPKPSDILRKKMASEIIHNYPDGQQIRKDELESMMFFAKKQKGTIDVWWLYDDGGLTILLPYILSTRSSWSDCKLRVFALTNRHHELKSEELCMASLLSKFRIDYSSLTMIHNITDPPETKTQQFFDSLIKNFRNNNGETVIEDAELESLRERTNRQLKLRELLLKHSNDASLIVMSLPMPRKGMVSAGLYMAWLEALTRDMPPFLLVRGNQQSVLTFYS |
| *NlAPC14*  (*Nlug19381-TA*) | MDKASVEVNMSGEILQNKETFNEQSAVEQELQDLMNSDKVPSRFKVARVSVSELLPNRDVNRLTPTIYENSSYDSRHSRSLNQLTREVLPRAEHYKDITSVHHAQRPTLEELHEARFLEKDQRRKKVEGVVEEDKSGANHKKGKVIKFGWVEGVYMRCLLNIWGVMLFLRLSWVIGQAGVGMGFAVIFLANIVTLATTLSMSAVSTNGLIEAGGIYYMISRSLGPEFGGAIGLMFTLANAVAVSMYIVGFCESVQDMLRLFGMSIIDNSTNDIRVVGVVTLVCILILAFVGMEWVTRTQMVLLIVLIASQIDFVVGSIMGPKTLTEMSKGFVGYDLEVFKKNLVEDYRYFEGVEHNFFSVFSVFFPAVTGIVAGANLSGDLKDPSKAIPRGTLLAILTTFVSYLSYALMMGGCVLRDASGNVTALREALDSGLAPPALYMTISQTCLGNNSAACRYGLHNDNQAMELVSVWGPLIYGGCFAATLSSAIASLVGAPRVLQALAKDKLYPFIYFFSKGYGANNDPIRGYILVFCIAFGCVLIAQLNAIAPLLSNFFLAAYALINFSVFHASISKCPGWRPAFKYYNAWVSLIGTLLCIAVMFLISWWTALVTFFVVVTLYLYVSYRKPEVNWGSSTQAQSYNFALRSALELNRVXEHVKNYRPQVLALSGDPGSRPAMIDFANLITKGNSLLVCGHILKGNHSQRVHDALTQKAQRWLDQHRIKAFYAVTSERTGGTFETSAEALMQLTGLGKLKPNTVLMGFKHNWHTCHLDELVNYFNLIHVALDSHLAVGILCLENGLDYSNYVQDKDVILISSLDKKDAEEKLRRNQSAGQLSLDETIAFIDGEQSDIAPAAAAVNTHPDPNGGDVTQQLDKDSESSVSESGGDLFVGPSGEPLAPNLLKDITRFRRKQDKGFIDVWWLFDDGGLTLLVPHILRTRSQFADSKLRIFSLASRKSQLDHDQKNLAQLLLKFRIDYSDLTVISDINKPARSETRLQFENLIEKFRLRGNDPAKADGSKIITDNELVLLHEKTNRHLRLRELLFEHSLDSTLIIMTLPMPRKGTVSAPLYLSWLETLSHGLPPTLILRGNQTSVLTYYS |
| *NlAPC15*  (*Nlug22218-TA*) | MSSGGTKKDPPAVGADWGSVELGDVNGERRARFQVNLVEKNKDDDGNETDRLCDGEEDYDEDDDHHGAYVKSFRHMTREALPRLDNYRNIMSIQAAYRPTLDELHNATLHHSNKGPGQASNGVHPGGSPDGGVVKFGWIKGVFIRNLLNIWGVMLFLRLSWVIGQSGVYDGCVIILASSVVTLITALSMSAISTNGVIKGGGTYFMISRSLGPEFGASIGLIFALANAVACAMNAVGFSESLLDLLKKNGVTLVDGGIQDVRIVGVITIFLLVCIVVVGMEWEAKTQMGLLAILLVAISDFFIGGIIGPTDENKRAQGFVGFNATVFATNWKPDYREYQGTKHDFFSVFAIFFPAASGFNAGANISGDLRDPGKAIPKGTILAIVVSTLLYVGMAAMIGALVVRDASGDVTEYLAGIAYNCSGRDCQYGLQNTVQVIELVSPFGLLIYFGCFAATLSSALACLVSAPKVFQALCKDNLYPYISWFGKGYGKNDEPVRGYILTFFIALGFILVGQLNAIAPLISNFFLAAYALMNFSTFHASLIKPVGWRPTFKYYNMWLSLVGAILCVGVMFLISSWTALITLAVVLALYLIVSYRKPDVNWGSTTQAQTYKTALTAVQQLSRVEDHVKNYRPQVLVFSGLPSSRPALVDFAYLITKNISLLVCGHVVSARVNNRTRTALRHKAHSWLHCHKVKAFYSEVDDVSLEEGGKALLQATGLGKLRPNVVLLGYKADWAQCDRLQLAHYFHLVHKSLDLYMAVAILRVPGGLDHSKQMSDEPEILALDEGNTSILNTRQNSQESIPRNVSYSQMSQVAMHHSMRSIYDPAQQTFVSDVSELTPPSTPNVSRSRKVNVRPDADSDQKKKDVKGDRNSRQSRGSVYNKVVGRLGTVHESTNSNNHPPTEQQTSPVSCRSIGGTELPKEVLHSITQFQRKRKKGHIDVWWLYDDGGLTLLLPYLIQTRSEWSNCKLRVFTLANKKDELQFEQRNMASLLAKFRIDYSDLKLIPDITKKPQEETTLFFENLIKDLKAPENDEEGKEEDQGYITEADLMATKDKTNRHLRLRELLLENSTDAELVVMTLPMPRKSVVPAALYMAWLELLTHDMPPFLLVRGNQQSVLTFYS |
| *NlAPC16*  (*Nlug17187-TA*) | MSLKKLVRVFSRRKTDDDELGDSKPQLARVLELVDLTALGVGSTLGVGVYVLAGAIARNDSGPAVTLAFLVAAVASAFSGVCYAEFAARVPKAGSAYIYSYVSVGELAAFIIGWNLILEYVIGTASVARGLSNYVDNLLGYVMKNTLTKLFPLHVSFLSPYPDFFSCGLILVQAMILSWGVKESTTLNNIFTAVNLLTVATVIICGCWKADVKNWMIKKEDIPAGVRGGEGGFMPFGVSGIMAGAAKCFFGFVGFDCVATTGEEAKNPQRNIPLSIVLSLIIIFLSYFGIATVMTLMYPYYLQDPDAPLPFVFNEVGLPSIKLAVTIGAIFALCTCLLGAMFPLPRILYAMSHDGLLYEFMSRVHPVSKTPVLATXLSGVFAGIMAAIFNLEQLIDMMSIGTLLAYTIVAICVLVLRYRDSNPVMYETPLMRRQNGMPSAKNQTNFIGNSVSQLFNLTMMKYPNSSTERISFWAITAMIVSLCCTCLCLIYMEPALASGESWAVGIVTIAIMVALITLLVLHRQPQSNTPLTFKVPCVPLIPALSIFMNSYLMLKLDMHTWIRFGIWLFIGMVIYVTYSIPNSVEGIRDRISAAERKPVQVGSNKSTRKNDNQKF |
| *NlAPC17*  (*Nlug17189-TA*) | MKYDNMLDKVKSVLAAMWTTLSRKKPLVSSYVEASDLCRVLGLVDLTALGVASTLGMGVYIMAGSVAKIAGPGVAFSFLIAGFAATLSALCYGEFAARVPKAGSAYVYSYVTMGEFVAFIVGWNLCLEYVIGTACVARGLSNYIDALVGDTISTFFSTYFPMNIPFLASYPDFFGFFIMFSFSCVVAGGVEISSTFNKLFTFINLTTLVIVIVAGAYKANFSNWTLSPTDEGFPTDGSGGRGGFLPFGFLGVFSGAAKCFYSYTGFDSVATTGEEAKNPQKLIPLALLMTLTIAVVTYLGVAISLTLMWPYFDQSVRAPFPYAFEKTNMPGVKWVVTTGALCALCSCMVGCIYATSRIIYSMANDGLLFKPLRRVNSKTQTPLTATLLIGAFTGMVATIFDMEQLVDMTSIGTLTAYTIVSFCILLLRYEVQGVTEEKGLTQTADTLAEGSTLLRAFFNMDKAATPTSQSSLIVKLMVAFFTLSSVAFSASVAGVSNATTRPFAVFLMVLSSAGIIASVLVIIRQPQSREQLSFRVPLVPILPIVSSFMNIFLMVMLNVQTWVRFFIWMLLGFVIYFGYGIRHSAEAKKREQSPPAYNMSNPPARFSVTVD |
| *NlAPC18*  (*Nlug20896-TA*) | MPGSRDKILSEVFSGICNKMNRCKPLSSDDVMATPLRRCLNTFDITLLGIGHMVGAGIYVLVGTVAKDQAGPGIIVSFLIAGLTSMLAALCYAEFGTRIPKAGSAYVYTYVSVGEFWAFVIGWNLILEHMIGAASVARAWSGYIDSMLGNVVSHTIMDTVGGLHEGLLGQYPDFLAFAFCLAYACVLGTGVKFSSWINSGLTLVNLIVITFVVVLGFYYADFDNWTKYGFLPYGYSGVIAGAATCFYAFVGFDSIATSGEEAKNPQFSIPVATILSLSLVCLGYVLVSGVLTLVVPYSEIEPTSALSSAFGSLHLPWAQVFIGVGALCGMTTTLLGSLFALPRCMYAMAQDGLLFSCFTRVNKATQVPLLNLAVSGILTAVIALLFDLEKLVEFMSIGTLLAYTIVSASVITLRYRPEILPLLSRENSSLSDLADSAPFANDYTQGRAGLLKTSGYLYRFLDPMLCESPPGVAVSTFVFIYCMFSALLCTHLRINGDPTMGANVVATVFMSSAMIASLVVIAAHEQNMTGLKMFRVPLVPWLPGLSVFCNVALMTHLNLLTWLRFIVWMALGLLVYFLYGMHHSKENITAGSSYSVLLSPSESNKPKWGSIQTPTKQDPDTESEKNLQ |
| *NlAPC19*  (*Nlug21374-TA*) | MVHNWNIIKIRSDRLLRIFARRKTDDEDLTSGEAKPQLARVLGLVDLTALGVGSTLGVGVYVLAGAVARNDSGPAVTIAFLVAAIASAFAGVCYAEFAARVPKAGSAYIYSYVSVGELAAFIIGWNLILEYVIGTASVARGLSNYVDSMLHYTMKNALIEAFPINVSFLSPYPDFLSCGLVLIMSLVLAWGVKESTRLNNAFTGVNLLTVATVIVCGCWKADVNNWLIKKENIPANVKGGEGGFMPFGIPGIMAGAAKCFYGFVGFDCVATTGEEAKNPQRNIPLSIIVSLIIIFLAYVNLSFYLVILLLFXDPDAPLPFVFGEVGLPVVKIIVNIGAIFALCTSMLGAMFPLPRVLYAMSHDGLLYEFMSRIHPVTKTPVLATLLSGVFAGIMAAIFNLDQLIDMMSIGTLLAYTIVAICVLVLRYRDPNPVMHERPLMDIQNGMNGHKQKSDNNGQHQSAVQPDNDEVSELSY |
| *NlAPC20*  (*Nlug28084-TA*) | IGHMVGAGIYYWWEQWQRPGWSRNHNFVPDSRLTSMLAALCYAEFGTRIPKAGSAYVYTYVRAASVARAWSGYIDSMLGNVVSHTIMDTVGGLHEGLLGQYPDFLAFAFCIAYACVLGAATCFYAFVGFDSIATSGEEAKNPQFSIPVATILSLSLVCLGYVLVSGVLTLVVPYSEIEPTSALSSAFGSLHLPWAQVFIGVGALCGMTTTLLGSRFALPRCM |
| *SfAPC01*  (*Sfur05883-TA*) | MSDPSAFSINSSHEAPRIAISPGNESIPLITRNSFFRKITKLCGGRDMDSGNTENGYVEFGSLGEISNGRTLGTFAGVFSPVALSMFSALIFLRVGYIIGNAGLITTLVQFAIAYGILFFTVASICAISTNGAVEGGGAYFMISRTLGPEFGGSIGTLFFLANVVSSALYIVGFGEGLVENFGKTGYLMHEDSKFFLPDGEWWRFLYCTVLNVINLLVCLIGAGMFAKTSVAILGVVTVSLISVVVSLSVQSSFEVPIPEVNKLHVKANGTFTGLSVSTLMDNLYPSYAPDYTSGITTELTNFASVFGVLFSGVTGIMAGANMSGELRDPAKSIPRGTLSAVAFTLFCYATISLLIAASCSRFLLQNNYIFMLPTSVWPPLVTVGILTATFSASLSNLIGSSRVLEALAKDNVFEVWDLRTFHLLRTVSSLDHCDVIFAPTGTAIYAVSLEQESEEDSNYESSFKTLDACDYSSIAIPVKAFIELTVARSVREGLHHLVHLSGLGAMKPNTIVFGYYDDEIPQDYFLDADSPYRTVRFQQQSAADGGVVEGVDGSELFRLRRSADVDKQLTGEEYVSCISDVLRMKKNVCVCRHFHTLDKATVKTGKYKYIDVWPVNFFQPSAEDPFNTTSLFMLQLACIINMVRGWKHLQVRVFLCDSNTSVSSLTEFHAQRSTEQRFRHLLHSLRIKASIHPVAEWSTQLASLRGGSVFRTKDGEVPTSNVAKTYLSNTNQVMRHHSPDSSTALLLLYLPEPPSAKSTVDQRAQYLDYLNQLTAQLPATVLVHGISTVTSTTL |
| *SfAPC02*  (*Sfur05484-TA*) | MTITSRSVSAEKLRSPEEVPLQPSAAQSSAAGNGGDDGVCLKAKMSLLNGITVIVGSIIGSGIFVSPTGVLVSTGSVNMALVVWTASGIFSMVGAYCYAELGCMISKSGADYAYIMETFGPFLAFMRLWVECMIVRPCSQAIVALTFSIYVLKPFFPDCDPPEESLRLLAVCCILILTFVNCWDVKWATRVQDTFTYAKLLALCIIIGAGVLKLFEGKEKFNSFLYLVYQLEKSH |
| *SfAPC03*  (*Sfur05486-TA*) | MLMVQEFLKEEIDNSQEYKDLKLGSGEATSKYTIPKAIHSPKLPDDIHDELYNSISRSHLGPPPAFDQDIAKTELRTSSVPIPIKIIPMIFAVTFAGKTQYFTFENTTYEPTSIALSFYSGLFAYNGW |
| *SfAPC04*  (*Sfur05485-TA*) | MTNVAFYTTLSPEEVLKSEAVAVTFANEIFGYLAWTIPVFVAMSTFGAVNGILLTSSRLFYAGACEGQMPEILTMIQIKRLTPTPAVLCIVIK |
| *SfAPC05*  (*Sfur06843-TA*) | MTLWKTLSRRKSLTSICTEESQLGKVLTIFDLTALGVGSTLGVGVYVLAGSVSKDVAGPGVVLSFLYAAIASAFAGICYAEFGARVPKAGSAYAYSYVTIGEFVAFIIGWNLIMEYVIGGASVARGLSLYLDSLIDGRMEKYFRSIISIESDFLSEYFDLFAFAVCVFLGFSLAVGLKESMLLNNFLTIVNIGVVLFVIVFGAFKADVSNWKIPKAEVPDWAGEGGFLPYGIFGTIKGAATCFYGYVGFDCIATTGEEVKNPQRAIPLAIIISLTIIFLAYFGVASVLTLLLPYYLQDVNAPIPYAFSQVGWTFAQWTVAIGGTFGLFASMYGGMYPLPRIIYAMSNDGLLFKMLGEVHKKYKTPFIGTILSCVLTGIMAAAFQLKHLVDMMSMGTLVAYTIVSACVLLLRYRESNEPTEHKMQRSNEHTRLVNNSNEVTCCAVVKQLFNFGAVGEATKVTSFVASTQIVTFCILSILLSFCLVQWEQHLIEGSNMHIAMCLIIAGVMLVCLLSIFRQPSNTKGLSFKVPLVPLLPALSIFLNIYLMMLMDNDTWIRFTAWMIVGLFIYFGYGISNSVEKDAKVVRSVDYDSNGHWAADDDMADD |
| *SfAPC06*  (*Sfur06887-TA*) | MWAVLSRKKPLVSSYVEASDLCRVLGLVDLTALGVASTLGMGVYIMAGSVAKIAGPGVAISFLIAGFAATLSALCYGEFAARVPKAGSAYVYSYVTMGEFIAFIVGWNLCLEYVIGTACVARGLSNYIDALGGDRISHFFSSNFPMHIPFLAAYPDFFGFFIMFSFSCVVAGGVEISSTFNKLFTFINLTTLVVVIVAGAYKANFSNWALSPSDEGFPTDGSGGVGGFLPFGFLGVFSGAAKCFYSYTGFDSVATTGEEAKNPQKLIPLALLMTLTIAVVTYLGVAVSLTLMWPYFDQSVRAPFPYAFEKTNMPGVKWVVTTGALCALCSW |
| *SfAPC07*  (*Sfur06889-TA*) | MAARTHESGEVPVSFTVQHFTCFLQLNSLLRVFSRRKTDDEDLLGDSKPQLARVLELLDLTALGVGSTLGVGVYVLAGAIARNDSGPAVTVAFLVAAIASAFSGVCYAEFAARVPKAGSAYIYSYVSVGELAAFIIGWNLILEYVIGTASVARGLSNYVDNLLGYVMKNTLTKLFPLHVSFLSPYPDFFSCGLILVQAMILSWGVKESTTLNNIFTAVNLLTVATVIVCGCWKADVKNWMIKKEDIPGNVKGGEGGFMPFGVSGIMAGAAKCFFGFVGFDCVATTGEEAKNPQRNIPLSIVLSLMIIFFSYFGIATVMTLMYPYYLQDPDAPLPFVFNEVGLPSVKLAVTIGAIFALCTCLLGAMFPLPRILYAMSHDGLLYEFMSRVHPVSKTPVFATLISGVFAGIMAAIFNLEQLIDMMSIGTLLAYTIVAICVLVLRYRDPNPVMYETPLLSRQNGINGKTKNQSNRIGNSLSHLFNLTMMKYPNSSTERISFWAITAMIVGLCSTCLCLIYMEPALARGDSYAVSIVAIAVTATLITLLVLHRQPQSNTPLTFKVPCVPLIPALSIFMNSYLMLKLDMHTWIRFGIWLFIGMVIYLTYSIHNSIEGIKDRISAAERKPKPTTDKSIKNATTKF |
| *SfAPC08*  (*Sfur06916-TA*) | MDTSPVSGVGAASGLNAQGTHDYDTTIDYSVQLAACTHETGVCYAEFAARVPKAGSAYIYSYVRTASVARALSNYVDNFLSYAMRDALREAFPINISFLSSYPDFLSCGLVLIQSLILAWGVKESTLLNNIFTSVNLLTVATVIICGCWKADVKNWTIKKENIPPNVKGGEGGFMPFGIPGIMAGAAKCFYGFDPDAPLPFVFGEVGLPAVKVAVNIGAIFALCTSMLGAMFPLPRVLYAMSHDGLLYEFMSRIHPVTKTPVLATLLSGVFAGIMAAIFNLDQLIDMMSIGTLLAYTIVAICVLVLRYRDPNPVMYERPIMEIQNGMNGHKQKATTMGNINQLFNLTMMKYPNSTTELVSAWAITVLIIFIAGTCLCLIYFEEALAAGEAWALFAVIIMTLASLVTLYILHRQPQNNTPLTFKVPAVPLIPALSIFMNSYLMLKLDLHTWIRFGVWLFIGMIIYITYSIPNSVEGIKDRISAAERKPPKVPKRPTTDVTKF |
| *SfAPC09*  (*Sfur06920-TA*) | MELANRKMSVILAWGVKESTLLNNIFTSVNLLTVATVIICGCWKADVKNWTIKKENIPPNVKGGEGGFMPFGIPGIMAGAAKCFYGFDPDAPLPFVFGEVGLPAVKVAVNIGAIFALCTSMLGAMFPLPRVLYAMSHDGLLYEFMSRIHPVTKTPVLATLLSGVFAGIMAAIFNLDQLIDMMSIGTLLAYTIVAICVLVLRYRDPNPVMYERPIMEIQNGMNGHKQKATTMGNINHNLS |
| *SfAPC10*  (*Sfur16786-TA*) | LTIMTKQVTSPVSDTCLMTPLSDAAPDGDKVRMKKQLGLLEGVAIILGIIFGSGIFISPKGVIQEVGSVGLSLVVWSLCGVLSMIGALCYAELGTSIPKSGGDYAYIYEAFGPLFAFLYLWDAMLIFVPTTNAIMGLTFAKYVIDPFFPECPLPEVSVRFIAACAICFFTFMNCYNVKLTTNIQNSFMFGKTGALALIIIVGIASFFMNDVNNFDAPFERSTFSPGPIAVAFYSGIFSYSGWNYLNFMTEELKNPYVNLPRAIYISLPLVTLTYVLANVAYLAVLSPDEMIASNAIAVSFGYRTLGVLSWIMPLMVAMSALGGLSVHIMTSSRMCFVGARYGHFPAMLSHLNINKLTPMPSLVFLNILSLLMLCTSDIHLLITYSSFVESFFLLLSVSGLLWLRYKKPNMHRPIKVSLIVPITFVILCIFLVIFPVFSAPYEVLMGVLITLTGIPAYYFGVVWKEKPAWFTKTLDGLTAFVQKLFMSAKEEGVEGFD |
| *SfAPC11*  (*Sfur12447-TA*) | TFGNKMFGSLSWTVPVFVALSTFGGVNGILFTSSRLFLTGAQQGHLPAVLSYIHVTRCTPIPSLVVTCLLSLVMVTQSSNVFTLINYFSLVLWLSMAACIAALLWLRVSQPRLPRPIRVHTALPASFLLCSLFLITMSAVTEPLNA |
| *SfAPC12*  (*Sfur12497-TA*) | MIPKSGGDYAYINEAFGALPAFLYLWVALLVIVPTGNAITALTFAEYIVKPAWPHCDPPYEVTRLIATLITCLLTAVNCYDVKWVTRVQDFFTATKVLALLMIVGVGVWALLVDGHTENMRAPFQGSNTSPGALALAFYSGLFSLSIILLILVFSVFSYRNYLNFVTEELRDPYKNLPRAISISIPLVTVIYCLVNLAYFVILPKVDLLSSNAVAVTFADRALGGFAWSMPTFVACSTFGALNGAIFASSRLFFVGARQGHLPKAIALINVDTCTPVPSLIFLCIVTVVLLFIEDIYMLINYVSFVEALFTLISVSGLLWLRYKMPDRHRPIKVNILLPIAFFIICIFLVTLPVFERPQEVCVGLIITLSGIPVYYIFIEWNKKPAWIQKTSSQYTIQFYFIIIILGYKNETLFEIII |
| *SfAPC13*  (*Sfur13658-TA*) | MVFDAGCYTNTVSKCLTTLDLTSLGVGSCVGTGMYLVAGMVAKNVAGPGVVISFIIAAIASIFSGACYAEFGVRVPHTTGSAYMYSYVTVGEFVAFVIGWNMVLEYLIGTSACACALSACFDALTDGAISSLVTESVGTFFGRPPDFLAFVITLLMMLLMAAGVKKSLVFNNILNAINLSAWVFVMTAGMFYVNTANWSEHKGFLPYGWSGVFTGAATCFYAFIGFDIIATTGEEANNPKKSIPLAIVTSLIIILIAYVTSSMMLTLIVPYEKVDQDSALVEMFGQVGAYRCKLVVAMGALAGLSVSMFGSMFPMPRIVYAMAQDGLIFRTLAQVLPLTGTPVVATFGSGFAAALAALLISLEVLVEMMSIGTLLAYTLVSTCVLILRYQPHSTNLVDLLPESLRTPVKGSPSKETLSNGQVTYGNQLHPDQLRSALGGVPGQPASATTAPQQPPSTGLTATLTQQQQQQQQRIMVRRVTRSSPDSDDTFPGEEPDEFSMRDDQFLVADRSENKFYGTVHGGSSAGSGGVAGPTLGAIGRRLQAVTYLCPAIFPWVDSGPATEDSGMFVMKMVGILYILILIFDLIIVLGMGSDSSIVTFLVIFFLLCIIALLLIISRKPQNRKTLMFMTPGIPFVPAIAVIVNIYLIFKLSILTLVRFTVWMIIGLLVYFYYGIKHSSLEEEDDSQNIELSVTTDKPAPAQQNVNAEHETPAWDSASYHQPQQAASPYTSIQQPPTSASNNPPLFVSPQQFPTWDD |
| *SfAPC14*  (*Sfur03256-TA*) | KLSGSRIWKSLVRKKAMDKWRMEQSELRRALDLIDLTTFGIANTFGLGVYVLAGMGARLAGPGICVSHFIVGVVTTITALCYGELAAAVPRSGSVYNYTYVIMGEFMAFIIGYLQCMMSIIGVACLAGGLSDYIDNMIDNKLGDYFANVNIPVILADPNVFAAILIAICSLIGVCNEHISAWYDEKDDADLKNWSWERGDEDFPADGSGGSGGFFPFGFHGVLVVARSCVYAYGGFEGISCAVIPVF |
| *SfAPC15*  (*Sfur03255-TA*) | KLSGSRIWKSLVRKKAMEKWRMEQTELRRALDLIDLTTFGIANTFGLGVYVLAGMGARLAGPGICVSHFIVGVVTTITALCYGELAAAVPRSGSVYSYTYVLMGEFMAFIIGYLQCMESIIGILIGGVELTVTFQKLLTFLNLTTLTIIIVIGVCNADLKNWSWERGDEDFPADGSGGSGGFFPFGFHGVLVVARSCVYAYRGFECISCAGEEAKNPRKLLPLAILLTLIIASVSYIGVAVSLTLMWPYFDESITSPFPYALDMTGMHDVNLLGNTYTCTRVLYSVGLDGLLFKWLGKVNSKTKTPIVSTIATALLSGECPLI |
| *SfAPC16*  (*Sfur15752-TA*) | MKIHRRSYVKGCEKDYMVEKINLFNASLLEASALVARRLTNNDTKKKEAHPDKDEDEENDQNEKEEEEHFIPSKPPSSQNGGSAINEGDAQPTQNVELQRELGLFSAVSLILSVMIGSGIFVSPSTALQNTGSVGLSLVIWAVCGLISLLGALSFSELSTVVPRSGAEYSYFRVAFSKLHPFLGPIPCFLYAWVVVLILRPAEVAIIILTFTEYVYSPVVTLSGFKITAAYEILLKKLLSLLTLGTISYINFSSVKLFVKLQNVFSSFKIVACFVVILGGVYSLSAGNVSNLNTGFQGSKTSLRDLVLALYSGLWAYDGWSSVTVVAEEIKNPEKNIFRSILIGVPLVTILYFFMNVSYMSVLTIPEMMSASAVAESFGEKVMGNLSIIIPIGVALSTFSCSLSVQFGVSRLCYAAGREGHMMEAFSYIHVRKCTPAPAVAVQGLLTTIFILAGDISTLIEFASFLIWIFYGLAMIALIVLRFKKPLANRPYKVPIIIPISLAILSLILAAVPIIFNPQIQYICALIFIILGMCVYYPFVYLKYRLPFMDKLTYLIQVMLEVVPPPSKPDDVTPSETTMTPQPSTVSANSSAPISFH |
| *SfAPC17*  (*Sfur08819-TA*) | MAPRKPRRMSGFVRRHTYGLEMEPQFASSLNGRQKKEVDWSKYGLRTEQQDSSTLSWKNSPEEGGYGAGGHQPADVSELFAGEQNNEPWWKSQFFISQPVLFGVWDGVFTSCLINVFGVIVFLRSGWIVAEAGVVNATLMVLATVGIALISVLSAVGICERCRMESGGVYFLLAHVLGSRIAAAVGLLYVFGQAVGCALFVLGFGESVAGLFGLGNSVWAQRGFGSAAILLLGVINVAGVKWVVKLQFLLLLVLLLAGLDFAVGSFIKTDIKNGFDGWLTGKLSENALPSYSAGNNWFTVFGVFFPTITGIMAGINMSGDLRQPTTDIPNGTLLAVATTTGLYLMFVLVLGSTCTRTALRTDYMIAAKVSALHVMLLAGLYVSSMSSCLGAMYGTPRVLQSIANENVIPVIQGLGKGRGPNRVPIYSMAVVALVTMSFLLVGDINSLAPIVTMPFLMMYAALDYAYFALAQTFDLLHHREQRFRSTQSGRSYGSSPSLQHSDISNDLDSLFPERSHHRTYTSITSESSPTIEHQQQQHRQLDQQTASSRMVHSKRGYWYSSWCNRWLSLFGALLKIGMMFLVHWSYALANCGVVFVIWIYIGLANPAVKPGVAIHFRLLRWLHAVLLRLCGKRTVEFEEIVVPPDTPGLHLSPSQLTDENEDFANRQRFHQSATIRPIPDSDHAAYGDVQILR |
| *SfAPC18*  (*Sfur07586-TA*) | MREELEDRPRISTLLGSLANYENTIPSTPADPDAKPAAGGARMGTLVGVYLPCIQNIFGVILFIRLTWVVGTAGAVQGFLIVLTCCCVTMLTAISMSAIATNGVVPAGGSYFMISRSLGPECGGAVGMLFYTGTTLAAAMYIIGAVEIVLTYMSPSLSIFGDFTKDASIMYNNFRVYGTGLLMVMGTIVFVGVKFVNKFASVALACVILSIVAVYVGIFMNFNGNDKLKMCVLGNRLLKDIHIDQCNKTVGGELYNLFCPGNSTKSCDPYFLANNISIENGIKGLASGVFLTNLGDSFLEDGQYIAKSLDPEEINRLDRPTYNQVMADITTSFTLLIGIFFPSVTGIMAGSNRSGDLADAQKSIPIGTICAILTTSTVYLSAVLLFAGTVDNLLLRDKFGQSIGGRLVVANIAWPNEWVILIGSFLSTLGAGLQSLTGAPRLLQAIAKDGIIPFLAPFAVSSSRGEPTRALLLTITICQCGILLGNVDYLAPLLSMFFLMCYGFVNLACALQTLLRTPNWRPRFKSLSFVGLSLCIAVMFMTSWYYALMAMGMAGLIYKYIEYRGAEKEWGDGIRGLALSAARYSLLRLEEAEPHTKNWRPQILLLAKLTSELVPKYKKMFTFTSQLKAGKGLCVCVSVVGGDYTRSSSDAITAKQNVAKMMEEEKVKGFVDVLISREISEGISHLIQTTGLGGMKPNTVILGWPYGWRQSEEEQSWHTFLHAVRVITASRMALLVPKGINFFPDSTQKMSGNIDIWWIVHDGGLLMLLPFLLKQHRTWKNCRLRIFTVAQMEDNSIQMKKDLKTFLYHLRIEAEVEVVEMTDNDISAYTYERTLMMEQRNKMVRELRLNKKESLGMVQAIVDHHHDLKTAKNQNQEQKSRTIRFQEPSEEDKQVIIDQKQNLFPILKTCIIHYKYEQNQTDGSSEKSPTPSNKSPAATNNAPSGDNPAITPDENNVRRMHTAVKLNEVISKRSYEAQLVILNLPGPPRDTKAEREHGVPRGVNRGLGACANGAGKWSRGHHHLLVKKHFCDHNISLL |
| *SfAPC19*  (*Sfur16670-TA*) | MMKASALAYAELGTMNTSCGAEYAYFMDAFGPLPAFLFSWVSTLVLKPSQLAIICLSFAKYVVEAFVTECEPPETVVKMVAIMSIMVILFINCYSVNLATGVQNAFTAAKLVAILIVICGGGYKLFQGNTQNFENMFSGETASIGSIATAFYTGLWAYDGWNNLNYVTEEIKKPSKNLPRSIMIAIPLVTLCYVLINISYLAVMSAAEMEESEAVAVTFGNRILGALAWLMPLSVTISTFGSANGTLFAAGRLCFAASREGHLMGILSYVHIRRLTPAPGLIFHSIIAAAMILSGNIESLIDFFSFTAWIFYGGAMLALIVMRYTRPNFPRPYKVPFIIPVVVLF |
| *SfAPC20*  (*Sfur14013-TA*) | MLYNAVEIATNCVMIVLQLSVCYAEFAARVPKAGSAYVYSYVSVGELIAFIIGWNLLLEYIIGVASVARGFSNYVDSVLGYVMRDTLTEYLPLKMSFLSPYPDFLSCAIILVLSLLLAWGVKESTLMNNIFTIVNLITVITVIFFGALKADLKNWSLHDVPQQAGKGGFMPFGISGVMAGAAKCFYGFIGFDCVATTAEEAKNPQRNIPLSIILSLVIIFLSYFGIATVLTMMYPYYLQDPYAPLPYAFDKAGMPAIKVIVTIGAIFALCASLLGSLFPLPRMIYAMAIDGLLFRKFALIHPKLLTPINATLLSGFIGGIMAMIFNLEQLIDMMSIGTLLAYTIVAMCILLLRYRDVNPIGSFEQKRKDKSVVVLGKLVCLFNLTSTKHPSSTSEKIVNWSIAFLIASLAAVSICLIHLENQIVAGDFWAITLLSTITAIILIILLVIYRQPQNDTSLSFKAPGVPLIPALSIFMNTFLMLKLDVHTWIRFSIWLFLGMIIYVFYSIPNSVEGLKDRLLYENQANFEIKGKSLNKSDNITTKL |
| *SfAPC21*  (*Sfur14014-TA*) | MHYTTIKRSDGCSSAMRSDGLVRVLTRRKTDDRLEEGGGATPKLARVLGLFDLTALGVGSTLGVGVYVLAGAVARNDAGPAVTLSFLVAAVASAFAGVCYAEFAARVPKAGSAYVYSYVSVGELIAFIIGWNLLLEYIIGVASVARGFSNYVDSVLGYVMRDTLTEYLPLKMSFLSPYPDFLSCAIILVLSLLLAWGVKESTLMNNIFTIVNLITVITVIFFGALKADLKNWSLHDVPQQAGKGGFMPFGISGVMAGAAKCFYGFIGFDCVATTAEEAKNPQRNIPLSIILSLVIIFLSYFGIATVLTMMYPYYLQDPYAPLPYAFDKAGMPAIKVIVTIGAIFALCASLLGSLFPLPRMIYAMAIDGLLFRKFALIHPNCFPSCCFHMSNSSREPDSGRRFLGDNTTINYHGNHSYNFAGNLQAAPE |
| *SfAPC22*  (*Sfur13707-TA*) | MSGRFNVQKVGLDNPGLTLDEGVIHSSPGHTLDVSGPDSGQRSADRKTSFTHYTLEALPRPEYYKSSGSGVKRPSLGELCGEEVVFSKETLKDPSKLNGRDCEQTDDGNQENEHTGIKLGWINGVLVPCLLNIWGVMLFLRLSWVVAQSGISGTLIIIAISSVVCIITTLSLSAICTNGEVKGGGIYYIISRSLGPEFGASVGVIFASANAVSVSMNTIGFCNSLNDLLAEHGIKIIDGADNDVRLIGVITICIMTVICAVGMEWESKAQNFLIVIIIVAIFDFLIGACLGPRTDMDRVKGFTGLSTEVFQENWGPSYRKSEGDEQNFFTVFAIFFPSVTGIQAGANISGDLKDPSSAIPKGTLLALLISMVSYVLFVLFAGAAALRDASGVVSEMLPNSTIHYWNCTDRVCDYGLHNSYTVMQLMSSNGNLIYAGCFAATLSTALTNLLSVPRLIQALGIDRIYPGLIFFSKGYGPHKEPYRGYVLTFVISTLFLMIAHLNVIAPLISNFYLASYALINFCTFHAGLVRPLGWRPTFRAQTYKTALMTAYKLSNSTEHVKNYQPQLLVLSGQPQSRPPLIDLANLITRNNSLMVCAEICKSRLNYKLRSIRMHKGMSWLSVRKIKSFFVVVDGVKFDAGARGLMQTSGVGRMRPNVLMMGYKNNWLSCANEELHAYFNTMHDAFENRLAVIILKMADGLDSSPFSEIDIDDTGSTLDLTVGDRGLIHNDSSSSLNLPLQQNVLSQTSLTISGETLFAEPNTGVSIYALILIISSLIFSYPDGQQIRKDELESMMFFAKKQKGTIDVWWLYDDGGLTILLPYILSTRNSWADCKLRVFALTNRQNELKSEELCMASLLSKFRIDYSSLTMIHNITDPPEEKTQQFFDSLIKNFRSNNSETVIDEAELQSLKDRTNRQLRLRELLLKHSSDASLVVMSLPMPRKGMVSAPLYMAWLEALTRDMPPFLLVRGNQQSVLTFYS |
| *SfAPC23*  (*Sfur13708-TA*) | MSSSGASKKAEAPPAGADWGSVELGDVNGERRARFQVNLVEKKTDEDGNETDRLCDGEDDYDEDDDHHGAYVKSFRHMTREALPRLDNYRNIMSIQAAYRPTLDELHNATLHHTNKHVFLLQKGRDGSIFQRRHIHKPPSPPPTPPPPPPPPHPPPPPHPPPPPPPXXXXXXXXXXXKNGPGQASNGVHPGGTQDGVVKFGWIKGVLIRNLLNIWGVMLFLRLSWVIGQSGVYDGCVIILASSVVTLITALSMSAISTNGVIKGGGTYFMISRSLGPEFGASIGLIFALANAVACAMNAVGFSESLLDLLRTKGVTIVDGGIQDVRIVGVITIFLLVCIVCVGMEWEAKTQMGLLGILLFAISDFFIGGIIGPTDDDKRAKGFIGFNATTFATNWKPDYREYQGTQHDFFSVFAIFFPAASGFNAGANISGDLRDPQKAIPKGTILAIILSTVLYVGMAAMIGALVVRDASGDVTEYLAGVAYNCTGRDCAYGLQNTVQVIELVSPFGLLIYFGCFAATLSSALACLVSAPKVFQALCKDNLYPYISWFGKGYGKNDEPVRGYILTFFIALGFILVGQLNAIAPLISNFFLAAYALMNFSTFHASLIKPVGWRPTFKYYNMWLSLLGAILCVGVMFLISSWTALITLAVVLALYLIVSYRKPDVNWGSTTQAQTYKTALTAVQQLSRVEDHVKNYRPQVLVFSGLPSSRPALVDFAYLITKNISLLVCGHIVESRVNNRTRSALRHKAQSWLHCHKNFGRQVKAFYSEVDDVSLEEGGKSLLQATGLGKLRPNVVLLGYKADWADCDRQHLKQYFHLLHKSLDLYMAVAILRVPGGLDHSKQLSDEPEILAIDEGNTSILNTRQNSQESIPRNVSYSQMSQGWYMAMHHSMRSIYDPAQQTFVSDVSELTPPNTPNVNRNRKVNVRPDAESAIAKKKDAKADRNSRQSRGSVYNNRLGTVHETTNNNANQRLATDAGQLTADQRSAVDAGQVVTSGSPVSCRSIGGTELPKDVLHSITQFQRKRKKGHIDVWWLYDDGGLTLLLPHLIQTRSEWSNCKLRVFTLANKKDELQFEQRNMASLLAKFRIDYSDLKLIPDITKKPQEETTAFFESLIKNLKAPENDDEANDAEDQGYVTEADLMATKDKTNRHLRLRELLLENSTDAELVVMTLPMPRKNVVPAALYMAWLEVLTHDMPPFLLVRGNQQSVLTFYS |
| *SfAPC24*  (*Sfur17005-TA*) | MHYVAFLYAQLNAIAPLLSNFFLAAYALINFSVFHASISKCPGWRPAFKYYNAWVSLIGTLLCIAVMFLISWWTALVTFFVVVNWGSSTQAQSYNFALRSALELNRVAEHVKNYRPQVLALSGEPGCRPALIDFANLITKGNSLLICGHILKGTHSQRVHDALTQKAQRWLEQHRIKAFYAVSSELGANFETAAEALMQLTGLGKLRPNTVLMGYKHDWQTCPTQELHNYFNLIHAALDSYQAVGILCLENGLDYSNFVQDKDVIVISSLDKKDAEEKLRRNQSAGQLSLGEIPFIDDINQQQLETSTGAAVNTNPDPNSSDVAQLDQDNNSSVTESQPASDLFLGPSGEPLAPNLLKDITLFRRKQDKGFIDVWWLFDDGGLTLLVPHILRTRSQFADSKLRIFSLASRKSQLDHDQKNLAQLLLKFRIDYSDLTVISDINKPAKGETRLQFENLIDKFRVKGNEPSQDENKRITDNELVLLHEKTNRHLRLRELLFEHSQDSSLIIMTLPMPRKGTVSAALYMAWLEMLSNGLPPTLILRGNQTSVLTYYS |
| *DcAPC01*  (*rna1244*) | MPWTTFRIWPFGNFGDGPDDQPFVSDKDKMSGYETNLYLYSEEMEDRPRVSTLLNSLANYSNTITPAPADPDAKPQAGGARMGTLIGVYLPCVQNIFGVILFIRLTWVVGTAGALCGFFIVLTCCCVTMLTAISMSAIATNGVVPAGGSYFMIGRSLGPECGGAVGMLFYTGTTLAAAMYIIGAVEIVLVRIMAGSNRSGDLADAQRSIPTGTICAILTTSFVYLSCVLLFAGTVDNLLLRDKFGQSIGGRLVVANIAWPNEWVILIGSFLSTIGAGLQSLTGAPRLLQAIAKDGIIPFLAPFAVSSSKGEPTRALLLTILICQCGILLGNVDFLAPLLSMFFLMCYGFVNLACAVQTLLRTPNWRPRFKYYHWSLSLLGLTLCIAVMFMSSWYYALLAMGMAGVIYKYIEYRGAEKEWGDGIRGLALSAARYSLLRLEEGPPHTKNWRPQLLVLAKLTNDFVPKYRNLFKFVSQLKAENIWDSFMYSGQYIAKSKLPSEVDVLNKPTYNQILVDMTTSFTLLIGIFFPSVTGIMAGSNRSGDLADAQRSIPTGTICAILTTSFVYLSCVLLFAGTVDNLLLRDKFGQSIGGRLVVANIAWPNEWVILIGSFLSTIGAGLQSLTGAPRLLQVQAIVDQHHDTNKGVTKVRFEEPNQNPNESRGDVEKQTKLDVGDHTENDQTDAEKTVNEVMKDIVTNKTSILDEDTKSSLTPDEGNVRRMHTAVKLNEVIVNKSHEAQLVILNLPGPPKETNIERESNYMEFLEVLTEGLERVLMVRGGGREVITIYS |
| *DcAPC02*  (*rna2264*) | MGFFKSLSRRKTNIDQGPLGESEVKLARVLGLTDLTLLGVGATLGVGVYVLGGSVAKNLAGPAVIISFLAAAIASFFSGICYAEFAARVPKAGSAYIYSYVTVGEFIAYVIGWNLILEYSIGTASVAKAMSDQLDALLGNVNRNVMTQVMPIHISFMAAYPDFIAAGIVFLLSILLAWGVKESTMANSIFTVVNLLVVATMMVAGSFKVDFANWVIPKESIPADKRGGXGGLAPGDLSGIMAASCPSASLGSSDVCYEMDCPPQAVLFALSTSMLGAMFPLPRVLYAMASDGLLYEFFSHIHPATKTPLLATLVSGVLAGIMAALFKLDQLIDMMSIGTLLAYTIVSICVLILR |
| *DcAPC03*  (*rna3122*) | MTLNGNVSANGVEVKSTEGSPIKVQMKRKITLLGGVSIIVGCIIGSGIFVSPAGVLAETQSVGLSIVIWTVCGLLSTIGALCYAELGTCISRSGGDYAYILVAFGELPAFLRLWVALLIMRPTTQAIVALTFAQYAIKPFFPDSELPDRAVLLLAAACLCKWSFFFLYLTEGSSNFDHAWDGNYDITKISLALYSGLFAFGGWNYLNFVVDELQDPYKNLPRAIWIAMPIVTLVYVCANVAYFTVLTKEEMLTSPAVAVTFGGKIYKELVWIIPILVAMSTFGGVNGILFTSARLFLTGSQEGHLPPLFSYIHIKIVFTISLFILIISNPSSLPQCLMSVLMLVTSDVFALINYMSVALWLSVGACTAGLISLRFTQPDLHRPIKVHLSLPIIFLACCIFLVVVPTIREPMNTVISLFIIASGVPVYYVCVKWKSKPALLLEMHGKFDRRFHFDWIFFGRILLHRMSYDIPCLLHIPGGRTHHTRTYEYW |
| *DcAPC04*  (*rna3123*) | MRLKKGLLTAINCYNVKWVTRLQDVFTATKMLALGGIVIAGMWFLAMGNTENIAHPMQNSNWDPGYVALSFYSGLFSYSGWNYLNFVTEELKNPYKNLPKAICVSMPLSTIVYLLVNVAYFVVLSKEELLSSNTVAVTFGAKILPMLTWLMPLFVSCSTFGALNGAIFASSRLFFVGARNGHLPKAIALINVKRYTPCPSLVFLCIITLLLMCIDDVFALINYATFVESSFTLTSVTGLLWLRLKRPDLKRPIKVDIILPITFFIIAAFLVTLPMYVKPWEVGIGLLMILSGIPVYMVFVYWKDKPRWLNNIADPLC |
| *DcAPC05*  (*rna4367*) | MYSYVTVGEFIAFVIGWNMVLEYLIGTSACACALSACFDTLTHGAISSSIKTSFGTIFGRPPDFLAFLITILMMILLAAGVRKSLLFNNVLNTINLAAWVFIMSAGLFYINTDNWNLHGGFLPRGWSGVFTGAATCFYAFIGFDIIATTGEEAHNPKRSIPLAILISLCIILAAYVTSSMILTLVGTLLAYTLVSTCVLILRYQPGSTNLMDLLPESLKTPIKGSPSKEYIANGQVCQTFLVALDLGSTLLAYTLVSTCVLILRYQPGSTNLMDLLPESLKTPIKGSPSKEYIANGQYPEQLKSSLKDIQPRPPPSPENPTDFQSDTLLQQQKQRIMVRRVTRSSPDSDDTYAEESEECSMRDDQYLVSDRIEGKFYGSVHGTGTAGGTSSNPLNIGPSLEQIQRKIHAATYLCPAIFPWVDTGPPTEESGILVIKLVGVLYVLIILLDVTMKLFLGSMGSITQFIVYAFIFAIIGILLVISRKPQNKKILMFKTPCVPFVPVIAITVNIYLILKLHYLTLIPELLSLDLDSNLAATYLCPAIFPWVDTGPPTEESGILVIKLVGVLYVLIILLDVTMKLFLGSMGSITQFIVYAFIFAIIGILLVISRKPQNKKILMFKTPCVPFVPVIAITVNIYLILKLHYLTLIRFTIWMTLGFIMYFYYGIKHSTLEEGSDNIELEATVVNSQIKVPKKAPAKENFEQYDMTEYDKRPTASSEPKTSYSKEDLFVPASAFPTWDD |
| *DcAPC06*  (*rna8297*) | MRVHQIRLDTQTVSGAFPLTFLDGMTNVYRNVQNVIVRIGPVVESAHSLLVNCHYDTVPDSPGTSKSKSTNDIIYSSKLSAGSSSKKNNNDSLLAPTYIFAVLIALFLLIYGFVMLIEKQLPQPLLLKNEAKYPGRFIAERAYNHLVNLTSLELQENLEEERLSLNAIYSSLSLMLTYIGSVMIPVMWLFSLSIFTVLREKTRLRANAPGILLNFFLFTTVPFLLCSYLIYCVYLLIIPIMGRSGSGNHAEEVVAFITTTIFSLLFNLLAPLILYVRSPKKILSLLSTGFLISVLLLVLTPLGFPYSGEISSPTPQRYMVMHVDRVYFSKENTVRERKSGLWLIDLDVNSPKTIENLLGDGLRLVNEETECSRELYCGLPYYLPVYSFIFQTHWIEDRTYTPLDVPLDLKLTYKHVEYSDGKKQTSDGRKPYSDEKSDADANNPSVGRERNATTAETESLNDEKADATRHSPARNATKGTTIKLSFALNGTDHVNIIASPYPGVELVRWSFLDGPPLKSKSQFRGRDTYYVFSTCASNLQTYHFWMEFFVPEKAFVDFGKAFDSVGGGKGGQADDRIYGGDIVDMAVAAHHVHGEKQMTKPLAAFFNKLPEWTVSTGWRLDFAWNKNGYVYHTRLDTTAQIPLATLQRTGDNILPLLLNLVNSKQLIAVHEYSEGSLIYFDFVGIFFVCITSTNTVLMCLTTLSIVLVGVYINAGSVRDALKTGYGRVLLRVIAFIIVNYLLCLLVNLAIAFALTKVNRAMAWYGSHIWLLFLYVIPTLVTSCVNILYFSHRHLSGFLLSRRVTEPSCFTPG |
| *DcAPC07*  (*rna8298*) | MDTPLRHWPYQSAIIGFRIHNFGNMTSIPILFSGLCYSEFASRISKSGSAYNYTYLSIGEFAAYIIGWNLVLEYIIGTASEAKAISNQIDSLMGNAYQKSMESLLPIKSSFLSSYPDFIAPIFVAVITGLLSWGASESAKVNNVLTFANLGTVAIIVFSGLFRLNPANWTIRREAIPPGVRGGSGGFAPFGVNGIVTGAAKCFFGFVGFDGIATTGEETRNPKRNIPLAIILSLLIVFVCYFSIAVVITMIVPYYEQNADAPFPAIFDRLGWPVMKWLVTVGSLFALFTAMFGAFFPLPRILYAMSRDGLLYERLSYVSSRTQTPLLSTALSGVITAVMSAVFKLDQLVDMMSIGTLLAYIIVAICVLLLRYSGDEVVVETCDSEVDFVHVDTLANTYKDMTREDSASTELTNVTDKSQLNIQRDKNQSSNQQQYLSVSHNQRPIQGHQKENAEIRGRADVVPVETQPNGNGVRNNPRSNLTQNMMTEFNTQSVLNGVTKTDNSPIGDVFYLPTNESFPDNAEFPGRNPNFPEGKSRKLRLSSLSASLRAETLKARLRRTCMTLFNTQGERTVTRTSQTVAKVCIYLLVGQTILLSFLLNGVTSGDREGAIELSAKGLVFRGINASRMGANDFVSANQNRSFADRIGDPLSVTGNGTNGLPNSRDQKPVSNVERLKEVGNAERLAFSYPVFSLRSRDDKTLAVTVPSIKCFVLLVLV |
| *DcAPC08*  (*rna8303*) | MSRLRNLYEALSRKKIDVGDDDVSIAEGKNPAGGADGVGKLPLERASDAPQLARVLGLIDLTMLGVGATLGVGVYVLAGSVARNQAGPSVVISFAIAAVTSLFSARVDLKSSVKPCSPKTIENLLGDGLRLVNEETECSRELYCGLPYYLPVYSFIFQTHWIEDRTYTPLDVPLDLKLTYKHVEYSDGKKQTSDGGKPYSDGRKPYSDEKSDADANNPSVGRERNATTAETESLNDEKADATRHSPARNATKGTTIKLSFALNGTDHVNIIASPYPGVELVRWSFLDGPPLKSKSQFRGRDTYYVFSTCASNLQTYHFWMEFFVPEKAFVDFGKAFDSVGGGKGGQADDRIYGGDIVDMAVAAHHVHGEKQMTKPLAAFFNKLPEWTVSTGWRAGLHLYTF |
| *DcAPC09*  (*rna8307*) | MWLSNFLDALSRKKSSYSEDAYDDGSGSREKLARVLGLTDLTLLGIGATLGVGVYVLAGSVAKNQAGPSVVISFIIAALASALSGLCYVEFASRISKSGSAYNYTYLSIGEFAAYIIGWNLVLEYIIGTASEAKAISNQIDSLLGNAYQKSMESLLPIRYSFLSTYPDFIAAGIVMLMSALISWGVSESTRLNSVFTFLNLGTVVVIVGSGLFKMNPRNWAIRKEDIPRTVRHGGTGGFAPFGLNGIIVGAAKCFFGFVGFDCIATTGEESKNPKRDIPLSILLSLFIVFVCYLAIAIVLTLIIPYYEQDAEAPFPHIFDSLGWPVMKWLVTVGSLFALLTAMFGALFPLPRILYAMSLDGLLYDCFSYVSPRTKTPVLSSLLTGALTAVLSAVFKLDQLVDMLSIGTLLAYTIVALSVLILRYSEDGADTPIKGTSCAEKETKSSSFAEKDINAKNFSVNALPEETLDPSVTYKETLDRSVTYEDIRPVGDEKSRRPKRMKSEQSGITLVKVYALQTEFLPCERDRELPRQKVGADNGEFNHENGGFKPENGRFNHENGGLNPENVGFNHEKVGLDNFPLGEKSAKSDEVNRVQLGAAQCVQIQRQTEENGREFSNRQQVSVSQPLSVTQTENGASVECEKQSSLLSESMRESNETLPADGSCEYNRVDNQVRTSYEAFPTRPEATKNPEALSVDGSDHARTYTTPVRSAAPVEINVRQTIATDLIVSANCTARVINESVCVRPEGKRNGAVSNIVVSETIQIGPLRSTLPGASSNTLCSDQFRTDACEHGNAWTRALAVLFNWNPAHAQVTATSQRIAKSCISLFVALTIAVCLMLNAVSSIDLSAEFVERNATVARSSGLSRNYLLSGRGEHDGYIDASTSEVKPFHANTLGTVIGMYFTDSQSALTGGPVPTSDCTSALQYATFTLLGLVYTVVFVCLARQNQNRARLKFKVPWVPLVPCLSIFMNIYLMINLDISTWVRFVIWLVIGFGIYFTYGISHSKQKLTKLA |
| *DcAPC10*  (*rna8788*) | MAVGDDKPGEEAEGPSDKMELMTPKKMTRFQVNKVDLSMSNPEELNKLWDDNVEDTTTNYAKSFRHFTREALPRLDNYRNIMSIQAVYRPTLDDLHEDNSVLPPCKTTSVPINDSLEDKESHKEQLGWFRGVYIKNLLHLWGIIFYLRLSWTLGQAGILEGMLIILSSSGLAFITALSVSAISSNGLAQGGGAYVMISRSLGPEFGASIGLIFSFANSMLTSMFIIGISELLVTSFSLELVDGGVQDIRILGVLIICVLLGCALSGVTLGYKVSIHSLFLGGAYVMISRSLGPEFGASIGLIFSFANSMLTSMFIIGISELLVTSFSLELVDGGVQDIRILGVLIICVLLGCALSGVTLGYKVRVVVFFVLAVSILNILLGSILSSIPYYTSSTSTNITSSTKEERGLKGFSLDVLQGNLFSSYSPGYNFFTVLGVYFPAIIGFLNGANSSKELKDPCCDIPKGTLYALLTGTASYVLVGLTSAAVMIRTVTPSTPYASSAAAGTPNVADMNSTSYALLPKYGLREYFEKLESNLNDLAPLISTPFLAAYALVNFSVFHASLIQPIGWRPTFKLYNMWLSLSGAILCMVVMFLICWITALFTLILILAMYLIIVYRKPDVNWGSTTQAQTYRSALEAVQSLNHVEEHVKNYQPQILVLCGLPSARPALVDFANLISKNISLLVCGHITKVKVPEVLRKSLYDKARHYLSVHKIKAFYTHVDEDEFDVGAKALLQVKVPEVLRKSLYDKARHYLSVHKIKAFYTHVDEDEFDVGAKALLQCTGLGKLRPNTLMMGYKTDWTHSKHELQKYFNVIHMVLDNYKAMCILR |
| *DcAPC11*  (*rna9421*) | MISGETKYFTFENSTTDVTSIALSFYSGLFAYNGWNYLNFIIEELKDPIVNLPRAIYISCTLVTVVYVLTNVAFYTTLSPAEVLNSEAVAVTFANRIFGPIAWTLPVFVALSTFGGVNGIILTTSRLFYAGACEGQMPEILTMIQVTKMTPTPAVLTIAFLSLLYLMSSNIFALINYVGFATWTFANRIFGPIAWTLPVFVALSTFGGVNGIILTTSRVGVNDNLFLLKPATHYQADLTFANRIFGPIAWTLPVFVALSTFGGVNGIILTTSR |
| *DcAPC12*  (*rna10173*) | MSCIFISPTGVVIPAGSVGLSLTVWVLCGVMYLHRSVGLSLTVWVLCGVMSMICVLYHILSLHSMFFTGIFISPAGVVIPAGSVGLSLTVWVLCGVMSMIGALCYAELGTSIPKSGGDYAYLFEAFGPLPAFLYLWDAMLIFVPTTNAIMGLTFANYVVKPFFHCENPPPDVAIRLIAASVICLLTFINCYNVRATTRLQNVFMFAKIAALVVIIIMGIGFMAMYGSPQFEDPWKNTETNVGKIAVSFYAGIFSYSGWNYLNFMTEELKNPYVNLPRAIYISLPIVTFIYTLANVAYFAVVPPALMSDAIAVTFANQVMGVFAWTMPFMVALSALGGLSVHIMTSSRMCFVGARYGHFPAMLSHINISRFTPTPSLVFLNILSLFMLFTSDVFLLITYSSFVESAFIMISVCGILYLRYTQPDMHRPIKVSLWVPISFVLICVFLVVTPILEAPREVGMAVLITLSGVPVYLIGVKWRDKPEAFTRSFNALTYFVQKLFLSALFPYIFTR |
| *DcAPC13*  (*rna12807*) | MLRLTGGSINHGGGEGYVEFGNIGEGASSGRTLGTFAGVFSPVTLSMFSALLFLRVGFIVGNAGLLVTLVQFIIAYGILVFTVASICAICTNGAVEGGGAYFMISRTLGPEFGGSIGTLFFLANIVSSGLYIVGCVEGLVENFGPSGLLVGEGNMLLPDSPGWRFFYCTLLNILNLLVCLVGATMFAKTSVVILATVLLCTSSSVFSFFVQDAMNVTIPDENHILHNITVNGTMEPVYRLYTGLSIDTLLSNMYSNYTRDYSTEDGVMNSFSSVFGVLFSGVTGIMAGANMSGELKDPGRNIPRGTLSAVAFTMLTYVLVSFLTAASCSRELLQNQYIFLMPINVWHPFITIGIVTATASASLSNLIGSSRVLQALAEDNIFGFITIIQDGQVFCLVSATCVTQYYSQNSGEELSFNKILYDNTVYAELTDIRSVQSFNLIQNSRGYGSPSRNGKRPENDARYNGNNVEATTIFKNNSNSTALNACKVVKDTTKNTVENGDIFDQPPNFRPSFKYFSWHTALIGLVGTLIMMFVINPIYSLCSIVLCVLLILLLHVFSPARSAEWGSISQALIFHQVRKYLLMLDSRKDHVKFWRPQMLLMVSNPRSSCPLIVFVNDMKKSGLYVIGHLNVEKRLNLFLSGMGAMKPNTIVFGFYDGDIPQDFFLSPSSPYATARFQGEGTEGEVFSLRGGGEKSVSPQEYVHMMSDVLRMKRNLCLCRHFSALDKQAIKSNVYRYIDVWPINFFNPSVEDPFTTSCLFLMQLACVINMKPSWKHLQVRVLLCDTRVRDPKTMALIQNKTYVLRGDGLLDGGIVANDVPEEQLLEDKRRNGFETRQDELYLAEPCGLTGVDSVDVFVEEDLGVTLYGGDCNDVPETRFRMCTGQAVVAYIPGFFAVKGLLVKIVVVISVYKSMLIVPMISGMTACTFVSLPDLPPQPTGFVPYLRNLTTLTNDLPPTVLVYGINAVTSTSL |
| *DcAPC14*  (*rna13147*) | MVGAGIYVLTGTVARDLAGPGIILSFMLAGITSMLAAVCYAEFGARVPKAGSAYVYTYVSVGEFWAFVIGWNIILEHMIGAASVARAWSGYVDSLCGGAISNFTIATVGELHEDLLGKYPDFLAFFVCLGYALLLGMGVKGSAFINSFLTLANLMVMAIVIVVGFYYGRLDNWINEGGGFLPYGISGVVAGAATCFYAYVGFDSIATSGEEAKDPAYSIPLATILSMSVVALGYILVSAALTLMIPYWTINPNAALPEAFSMLGLHWLKYTVTFGALCGMTTTLFGSLFSLPRCIYAMAEDGLIFKFLGKVNRTTQVPVINLAISGFFSAIIALLFDLEKLVEFMSIGTLLAYTIVSASVIILRYRPAITGLGSRDASHLTLTPEAVTPQSVSTLDFELGAGGTLKTGWQYNWMNCVLGRFEPGVCASLAVFIYICMCAGLCFHLHAVYQVDQRTSWWSLFTTTMFVFCMCACLFVIEAHEQNMEGLRFKVPLVPFVPGLSIFFNIELMANLNVLTWLRFIIWMALGLHNKAASKRWKTDQKSARYGMTVTSRGPCRTIPDNSDTNVKPRRTKRTNKWNGDYPTHDESSSSSEDESTPDRKKVTESEETVTSENKHENI |
| *DcAPC15*  (*rna15992*) | MTPAIKSSMRERDRLYIRYRHTGQQDTLQAYKAKKNATNYMVRKAQQNFARGILHGGPRSTWRKLNSVGLGNVKSLNPITADLDDLNDYFVSVVPSPCNSTKSRTLAELDTQTNLIQDNISFDFQEVTSQDVKKALTSIKSKASGYDGVDITMIRISLAWFLPIITHIFNKSLSSGKFPDFWKFSNIIPLNKVASPSSCVDYRPISLLPVLSKGLEKIVVRQIWAFIEEHNIIDPFQSGFRCHHSTATALVKVTNDIRWALDARQITILALLDLSKAFDSVDFDVLLKTLETMHFSNNVLSWIGSYLRDRKQRVFSHGKTSAWKPIRSGVPQGSVAGPLLFALYISSIQRVFVHCKYHIYADDVQVYIHCLPEDIAEAIRLLNEDLHNFSEWAKSLFLKPNPSKTQVLIVGTNNFVSTMASLQLPPVILDGQQIPFTQCAKNLGMKIDSTLSWSLRQRCKEEGILLTSHIE |
| *DcAPC16*  (*rna16797*) | MVVSRKNSKESSAGGKRESDIPLEEATEHSPIFETAEPGGQTRAEDTVCLKPKMTLLNGITVIVGSIIGSGIFVSPTGVLKETGSVNLALVVWTISGVFSMVGAYCYSELVLV |
| *DcAPC17*  (*rna16798*) | MISKSGADYAYIMTTFGPFLAFIRLWIECMIVRPCSQAIVALTFSIYVGAYCYSELGCMISKSGADYAYIMTTFGPFLAFIRLWIECMIVRPCSQAIVALTFSIYALKPFFPTCDPPDESVRLLAVCCICK |
| *DcAPC18*  (*rna17273*) | MIGSGIFVSPSGLLIRTGSVGMSFVIWISCGVLSLLGALAYAELGTMNPSSGAEYAYFMDAFGPIAAFLFSWVSSLVLKPSQLAIIALSFAKYAVEAFSTECEPSAIVVKLVSVLVILVILAINCYSVNLATGVQNVFTCAKLVAVAIVVCGGGYKLFQGHMENLENPFKGTTSRIGNIATAFYTGLWAYDGWNNLNYVTEELKNPSVNVPRSIVISIPLVTVCYVLINISYLAVMTSAEMIESEAVAITFGNRILGVMAWLMPLSVTISTFGSANGTLFAAGRLCFAAGREGHLLDILSYVHIRRLTPAPGLIFHSIIAILMIMAGNIESLIDFFSFTAWIFYGGAMLALIVMRYTRPNFPRPYKVPIIIPIGVLIISFYLVVGPIMDSPKFEYLYATIFIFSGLFLYIPMVHYNFKTILMDKFTTFCQLLLEVAPTQSVFEDYNIVTQKQ |
| *DcAPC19*  (*rna17952*) | MDFYPKDLISASRLLKSIENEYILYLALLTHIGNGVSAYWFSTRGNFALARCCNADPSPQHVAQGRTQDFVEGGASRVHMGVAHMVSGGKKRPSISQLYGEIPYNGDILSNRAIQRAPDGTSIADATGGAGSNTHGVKLGWIVGVLIPCLLNIWGVMLFLRMAWVVGEAGIGHSLVIIGISYVVCIITTLSLSAITTNGEVKGGECMSYSQKSQIYINRGDVLSCVVVCIITTLSLSAITTNGEVKGDGFFSRNTYQKRPSISQLYGEIPYNGVSGGKKRPSISQLYGEIPYNGVSGGKKRPSISQLYGEIPYNGDILSNRAIQRAPDGTSIADATGGAGSNTHGVKLGWIVGVLIPCLLNIWGVMLFLRMAWVVGEAGIGHSLVIIGISYVVCIITTLSLSAITTNGEVKGGGIYFIISRSLGPEFGASIGIILAFANAVAASMNTIGFCNSLNDLLASYHTKIIDGGVNDIRLVGVIAILIMVIICSFGMDWESKAQNVLLVLIVIAIIDFLIGAILGPQDNTSIAQGFVGFNSKFKLTFQDPGSAIPKGTLLALLITAISYVAFVLAAGGVAVRAATGNVTQLPDAFTPCDGLVDCAYGLQRSYTIMQLISSVGIIIYIGCFAATLSTALTNLLSVPRLLQALGIDRIYPGFIYLSKAYGRNKEPYRGYVLTFLISSAFVLIALGIDRIYPGFIYLSKAYGRNKEPYRGYVLTFLISSAFVLIGDNAFEYRLSVLVLRVPGGLDYSHTVDLPPDVDEKEDILYVKDKYPIHSNILQASSDYHLDRNSISAAEGVKLRELLPSKDLQSIQQYPSKKDETTNISARDHPGMNMMQCFDTKTKRGTIDVWWLYDNGGLTILLPHILSSRNSWSTCKLRIFAPARHGQDLEVEEQNMADLLSKFRIKYSSLKMISDISKPVQPESEQLFDSLIHQYRTHNPATLDVDLDAIQGKTNRHLRLRELLLEHSNDATLVIISRSLQGCFLMFDDVNWGSSTQAQTYKSALASAHKLMFVGDHVKNYRPQILVLCGQPQVRPPLIDLAHWFTKSHSLLICADVVKEKLSFKRRTNRTAAAIKWLQQNKDKAFYVLVDDGQFDDNVAALMKSAGLGKLRPNVIMLGFKNDWMTCPVSDLNAYYNVIQ |
| *DcAPC20*  (*rna20145*) | MANQLLQSLRGRSVHYELSVQLQRKGFIVGNAGLLVTLVQFIIAYGILVFTVASICAICTNGAVEGGGAYFMISRTLGPEFGGSIGTLFFLANIVSSGLYIVGCVEGLVENFGPSAAAFRPAKSKFDSVRKYLLMLDSRKDHVKFWRPQMLLMVSNPRSSCPLIVFVNDMKKSGLYVIGHVKAGKDFSELLADPTQDEYTHWLSLVDHLKVKAFVELTVAETVRSGLHHLVRISGMGAMKPNTIVFGFYDGDIPQDFFLSPSSPYATARFQGEGTEGEVFSLRGGGEKSVSPQEYVHMMSDVLRMKRNLCLCRHFSALDKQAIKSNVYRPSFKYFSWHTALIGLVGTLIMMFVINPIYSLCSIVLCVLLILLLHVFSPARSAEWGSISQALIFHQVRKYLLMLDSRKDHVKFWRPQMLLMVSNPRSSCPLIVFVNDMKK |
| *DcAPC21*  (*rna22275*) | MIPKSGGDYAYINEAFGPLPAFLYMWVALFVIMPTGNAVTALTFAQYILQPIWPHCDPPYSAVRLLAAVITCLLTAINCYNVKWVIRFYITCTYSSMFFISEFGGLYIENCVSYHMVISERFKS |
| *DnAPC01* (*rna-XM_015515484.1*) | MNTATTENVKITDSIPEETGDRVRLKKQLGLLEGVAIILGIIFGSGIFISPSGVMIEAGSVGVSLTMWILCGMLSMIGALCYAELGTSIPRSGGDYTYLFEGFGPLPAFLYLWDAMLVFVPTTNAIMGLTFANYVIKPFFPECDNPDDAVRLLAAAVICFITFINCWNVKATTKVQNVFMFTKISALILIIVCGGVYMYSNGFSKFMNPWQGSVTDPGRLAVSVYSGIYSYSGWNYLNFMTEELKNPYVNLPRAIYISMPLVTIIYVLANVAYLAVLTPHDMVTTKAIAVTFGHLAMGSFEWIMPLMVALSAFGGLCVHIMTSSRMCFVGARYGHFPTFLSYINVERYTPTPSLVFLNILSLLMLFTSDVGMLITYSSIVEAFFTMLSVSSVLWNRWKRPNINRPIKVSLWIPITYVIVSLFLIVLPCYVKPFEVGMGVGITLLGIPVYYLCVVWKTKPMWFQNSLKHVTFTIQKLFVSAKEEKAEDIWE |
| *DnAPC02*  (*rna-XM_015513055.1*) | MAWTIPLFVALSTFGAVNGILLTSSRLFYAGACEGQMPQILTMIQINRLTPTPAVICICLLSLIYLQISNIYALINYVGFATWLSIGVSVLCIPVLRFTQPDLKRPIKVNMFFPVIYIAATLFVTIIPIIASPVETGYGCLMILTSIPVYGVFIFWKNKPMIFHKIVGCLTRYLQILLMVTTSKTPAKV |
| *DnAPC03*  (*rna-XM_015512883.1*) | MGAKRKEGGSEVEPDVAPAENDKIELKPKMSLLNGVTVIVGSIIGSGIFISPTGVLENTGSVNASLIVWILSGVFSMVGAYCYAELGCMISKSGADYAYIMETFGPFIAFMRLWVECMIVRPCSQAIVALTFSLYVLKPMYTDCDPPEESTRLLATCCIGSDLGVWWPLAGMHCGAPYT |
| *DnAPC04*  (*rna-XM_015515208.1*) | MAVGKKLNRKKTMAGVVRESSMLDRVLTTTDLTALGVGSTIGVGVYVLPGALSKYVAGPAVVVSFFIAAVASVFAGLCYAELSSRVPRAGSAYSYAYIAVGELAAFIVGWNLLLEYTIGGASIARGMSLYIDSLTNKTMETAFRELYEIELPYLSEYFDFFAMFIVIVFSVALACGLKDSVRLNNLFTLLNCAIMILVIVGGSFHIDFKNWSLPKSEVPNWAGEGGFWPYGLQGALQGAATCFYGYVGFDCIAASGEEVKNPQKSLPLAIILSLFIVFLAYSGVSAVLTLMIPYYAQDANMPLSHAFDVIGWTPLKWIIGVGAVFGMCACMFGSMFPLPRILYAMSNDGLIFKSLGKVHPRFKTPFFGTIFAGIITGFFSALLNLQQLVDMMTIGTLLVYVMVAVCVLYTRYQEQSDMDYDILADEYIESTALVTIKVKYTKKQILKQLFNFHKFVRANSLSSYVASLQTTCFTITCFPLGLYLSHWYELNGTHWIIVQVIVGVMILQLVSIAMQPTSKTPVAFKVPLVPLTPALSIFINIYLMFFFDIYTWTKFIIWMIIGFTIYFGYGITHSKENNPEINIVNSQSKSSLNID |
| *DnAPC05*  (*rna-XM_015523958.1*) | MPGSRHKILSHVFSGFSEKMGRTKVLDIGTQAIETPLKRCLTTFDITLLGIGHMVGAGIYVLIGTVAKEMAGPAIILSFMLAGAASMLAALCYAEFGTRIPKAGSAYIYTYVSVGEFWAFVIGWNIILEHMIGAASVARAWSGFFDSMFDNVIRNTTISVLGELHETLFGKYPDVFAFFVCLLHACILGVGVKTSSYMNSFLTLINLGVMAVIVVAGYYYGNSDNWSSDGGFMPYGTTGIIAGAATCFYAYVGFDSIATSGEEAKDPAYSIPVATIIAMSVVCTGYVLVSGALTFLVPYWSIVPDAAFPAAFAGLDLNWIKYLVSVGALCGMTTTLFGSLYSLPRCIYAMADDGLIFKFLAKVNKKTQIPIINLAISAFLCALIALFFDLEKLVEFMSIGTLLAYTIVSASVIILRYRPTNRGLVRDSSSLLELPTSQADSFEMDMGGRLKPSYKFLEPFFGEFEPGYIVCVSIGCFTSSTVILCVYIQYWFNIEHVSWTDGVVLAMLGTDLILCLLLIEAHEQNSTELPFMVPHVPLIPSLSIVCNIVLMTNLNLLTWIRFFIWMVXGLLIYFLYGMHHSKENDVTSYSVLLSSSEAGKTPWGAINKSRKRVKSEDDRKPIIDNEELAENGYYH |
| *DnAPC06*  (*rna-XM_015511330.1*) | MTSIPKNVKLRRELGLFSAVCLIISVMLGSGIFVSPANALKNTGSVGMCLVIWMSCGLLSLLGAMSYAELGTVVNKSGGEFSFYQSAFADMHKFWGPLPSFIYSWVSIMYVRPAEVAIIILTFAEYFIRPFSIMTSMTPENEHMVKKTVSILALGIITFINYTSVKCFIKIQNVFTICKVTACLVVIGGGLYQLYQGNTKNLMTGFEGTTLSLDSLPIAFYSGLWAYDGWTATTVVSEEIKNPQRNILLSILLAVPFVTMIYVLMNVSYXTVLSVTEMTSVHAVAVEFGTRALGSFSFIIPLGVATATFGCALSVQFGITRLCFAASREGQMLEVFSYVSVKKLTPAPAVVLQGILTLICLLCGDIVVLIEFASFLVWMFYGISMAALLVMRYTKRDVKRPFKVPIVIPIFVLIVSTILFITPILNDPKPQFLIGLVFILSAFLIYIPFVYQKKRLSIVDNFTKFIQVLMVVVPPEKDEANSEENRVVEDDEGETEVPAIAALV |
| *DnAPC07*  (*rna-XM_015511525.1*) | MAELRNYKNDQSFMKTILRRKKESDLSTEPMKNQLSRVLGLQDLISLGVGSTLGLGAYVLAGEVAVKFTGPAVVLSFAFAAVASALSGLCYAEFASRVPKAGSAYAFSYVGIGEIVAFLIGWDLILEYSIGCASIARALSGHIDKPLGYPMRSFFIKTFPMNVEFLAPYPDLFSFTSILLLTLLIAWGMKESSLLNKIFTVVNLLTVITVVMTGLYKIDISNWSLPKEKIPLNIEGGEGGFLPFGWSGVFVGAATCFYGFVGFDAIATTGEEAKRPTRDIPLAIVISLSIITLSYCSVATILTLMWPYYLQDPEAPFPHIYQQLGWHALEWIVTIGAVFALCTNMIGTLFPLPRILYSMASDGLLFHIFSKVDPXTKTPFWGTFICGAFAAILSSLFDLQQLMNMMSIGTLMAYSLVCICVLILRYTNDNPEECKIRDNGRFRVSLMRLLSSSFNLPKSQITTKNTGRTSIIIILVYXVVSVCFCSSVSIVQIEGKFNMVTYVASIVSGVSLLVLCYSLSRQPQSTNRPTFHVPFVPVVPCLSVVLNIYLMTQLDTSTWIRFTVWLFIGLLIYLFYGLRYSVERLNQRRMEDETYLKQIRYEIQVY |
| *DnAPC08*  (*rna-XM_015511687.1*) | MNIDGIPTKLDNMNIDCVNTKSDNMNIDCTESGNMNINGVKTENMLPSKLKKSEPYDIFKQLPLEIINKIVLQLDFNDVLNLKLVNKMWRCVYVNQNEIWARICEDLNIRVIDYSRCLNDRSRHDSECIGYADVASEKLFGPICDYWQTFNRYIMIVKNIKNNDFPTIYIPRQHVEQSYCTDDYIVNINCQHKQPIQIVILNGANKPLKKKFLPIFNKFEELIKLRKYPLKVIGNKRYLVFEICSIIFVYSISKTEFTKKFFKVIQKSVDYGLNKDDFNEEFLNSHCDTKFDLYDHKLAMVHPAISTLFVTDLSTGKTYKELEFSSRGCIVDSMKCSDYRLMIGITKTKKKDLKTEHLAIVYHMKGCTQNNRLMIPLLGPVTQFKVTSNCIGVENTGSATPFITKQNNSYLNVFWLECDTFSFDCTRKYIYYNVNQSIFQYDLLKSMLSKFEVVQKIAIDAISNLLPLTPINDRYLLVRSTYPNSYDIFDVKEHVSVRSIQLTAGYSLVHVGKLSIMFSNSSEFMVIAFN |
| *DnAPC09*  (*rna-XM_015511781.1*) | MTEQADVKLKTYHWTNQIQSYEKLVRDEESKLNVDQGIKLGWIEGVLNPCLLSIWGVMLFLRMPWIVGQAGIFDSILIIFISLVIIIITAFSLSAISTNGRVKGGGLYFIISRSIGPEFGASIGILLALANTILVALNTIGFCLSLKSLLNTFNIHAMDSNFIFILIGFIAILIMGVLCGVGMDDEAKIQNILLIFIVGAIFDVLIGSFIGPTNDDAIASGFTGFSMKTFKENWYSNYTNDQSFFTIFAVFFPSVTGIQAGANISGDLKDPSSSIPKGTLLSILITITSYVVLVIVPGAVQLREASGHSNEILNEFYLNCSFRKCNQGLYHNENLMQTISLWPYLIYLGCFAATLSTALTALIAVPKILQRMGQDDIYPFLKYLSKGYGKSNEPYRAHILAIVISSIFLLIGELNAIASFISTIYLCAYALLNLCTFHVAHFQPLGWRPSYKFYNKWLSLTGAIICFLVMIFIDKQMSVIVACIIWILYTIASGKKDDINWGSSRQIQXIKTVIKNVYMADTIQQHVKNYIPNIMVLSGDPESRKELVYFAHIITKNNGLQMCINIIKDPLINKQKKELLEKGVNWLHQTGIKSLYNVLDNIDLDIGVHIINSCGHGILTPNIILIGYKHGWFNCTDDDIQTYLNILNLSNMDGIATIIVRLPINDTIIESKDRKLAKSTETQFYEDEKVNSTSLINEEEKYISKEFDCSVKMHDTQFSFLTKRNNGTVDVWWLFDDGGLALIIAHIFKSCDVWKKCNFRIFGVTDQLINVDMEKNKLKQLLAMYRVQFDFVDVVLAKATSLKTMANFTTLWNQQFINQESQPQDDEKYNKQTVIDTLYVKDLLETYSMQSDLIIISSKTTEKLDQIHMYWMEVITRGLPPCILIQGNKIKTVTASA |
| *DnAPC10*  (*rna-XM_015511765.1*) | MSEHDEIKNKSRPWIDQIQSYNDLYREEENKIELSPGIKLGWVKGVLIPCLLSIWGVMLFLRMPWILAQAGIFHSIIIIFISLLIILITTFSLSAISTNGKVKGGGLYFIISRSIGPEFGASIGILLALANTISAAMNAIGFCVSLRSLLQSRHISIIDSNVRFKALGVVSIIIMSILCCIGMDREAEVQNALLIAIIIGIFNVIIGSYNGPKSTLAKASGFTGFDMKTFKENWYSDYRVDNNVQQSFFSIFAVFFPSVTGIQAGANISGDLKDPSDSIPKGTLLSIIISIISYIVLIVVPGSVQLREASGNENEFQDGYFSNCSFRNCTKGLYKDINVYQSISLWPITIYFGCFGATISTALTALISVPKLLQRMGQDDVYPLLKYLAKGYGKSNEPYRAHIFAMIVSSIIVIIGELNDIASVISTIYLSAYALLNLCTFHVEYFKPLGWRPTYKFYNKWLSLAVAIICISVMIFIDSQMSLIIGSAICVLYILAGRKKEVLNWGSSMQTQQIKTVIKNVYKADTIQYHIKNYLPNLIVFSGNPESRKKLVSLAHLITKNNGVQMCVNIEKISITPRQKQICLDKGIQWLRKSGIKSLYVIIDNIELDLATYLIYSCGHGQLRPNIVMVGYKSDWLNCPYQDLQTYLNIFNVANMNNMSTIMVRVPSTEIHDDQNLLIQDFKHLNKNEEVPCKTQLDKNMRNQQKVNDCFVVNMKNKEFSFEKKKRNHGTVDVWWLYNDGGLSLIIAFILKHSTAWKNCKFRIFGVTNKVECLPEEKNKLKQLLSLYRINFDYLDIILSSTAGPTTMIYFSSLLKRATSKENQFEDFNLQKEYIAETLFLRDLIELHSFNSDLIILTTPKNNEEINLLFMCWIETISRGLPPCIIINGSTQSVLAVNA |
| *DnAPC11*  (*rna-XM_015513199.1*) | MMDDPWYKSTDSLTWALTRKKTDLDESNKKKLNRVLTFFDLTALGTGCTLGSGVYILAGTVAKSIAGPAVVLSFVIAAVVSAFSGLCYAELAGRVPKAGSAYIYSYVAVGEFTAFIIGWNLIVEYLIGTAGTAKAMSNYCDSLLGNPQKRYMTKYFPIHISFLGDYPDLTSFFVIVIIALLVAWGVRESSITNNIFTALNLITVCTVIVTGCYKANISNWSIPKSEIPPEVKGGEGGFLPFGWVGVATGAAKCFYGFIGFDSIAMTGEETKNPKRDIPLAIVASLFLSTIAYCGVAIVLTLMWPYFLMDADAPLPVLYENLDMPMIKIIVSGGAMFALCTSLLGTLFPLPRILYAMANDGLLFKFLSNINATTMTPLISTIISGLFAGTLAVVFNLEQLIDMASIGTLQAYTIVCICVLILRYTNNNPPIQDNNTTKSNGITVFXWLNLSNAKVPNSDTQYVSRALIFIFSVSTFVFGISLANMGSYHGTTRNVLMIINFVSILVLLITLIMLSRHPTAEEDLSFKVPLVPIVPCLSIILNVYLMMELEYKTWISFTVWLICGLLIYLFYGIGHSLEGNKQNIHQNTIQINLS |
| *DnAPC12*  (*rna-XM_015514996.1*) | MSVPKLPTDFRRDGLALITRMVRTKDLDDLQGESSPTGRFDPHHPTKLKKCLNTIDLTSLGVGSCLGTGMYVVTGLVARRFAGPAVILSFIIAAIASLFSGACYAEFGVRVPNTSGSAYMYSYVTVGEFIAFLIGWNMVLEYLIGTSACACALSASMDSLTNGAISASVQNYVGFLGKPDILAAGITLLMMVLLAAGVKKSLMFNHLMNAINLAAWVFLMSAGLFYVNLDNWTKNDGFLPNGWGGVFKGAATCFYAFIGFDIIATTGEEAHNPKKSIPLAIMASLVIILVAYVSSSIILTLIVPYTKIDENAALLDMFVQVGAPRCQMVVAAGAMAGLLVSMFGSMFPMPRIIYSMAQDGLLFKSLSQIFPLTGTPVVATVLSGVASAIAALVINLDTLIEMMSIGTLFAYTLVSTCVLILRYQPQTSTVIHFFPETMRSPMNAPKQIVTNGRVNFVVQDDQKYAYTNQGYGNQTSFYQTQLPLPQQIIPSHQSQRIMVRKVTRSSPDSDDTYFGDDSEEGRDDQYLVSDRCESKFYGSVHGGSTAGSTAAAGGFSAASANITRSIKAATYLCPAIFPWVDMGPATEESGMVVLKLVGVFYVLIIVFDVLLVFVSSESSTFVYILLYALLIAVIVVLGAISRKPQNKQILVFKTPWVPFVPSFSIAVNLYLIFQLSSMTLLRIIVWVSIGLFVYFYYGIKHSTLEPRVDEDERIELKMKSQTMTKQQNNRPQAPASAPAKSSANANTSSNANTTTPKXSASTGVASATTAAVVDEKTEPKRPTNLEPLPAKNDSNLF |
| *DnAPC13*  (*rna-XM_015515904.1*) | MSDHEEIKKKSIPWIDQIQSFSELYHRGEENKVELSPGIKLGWIKGVTIPCLLSTWSVMMFYNMPFILGQAGILISIIIIFLSMLIILFTNFSLSAISTNGKIEGGGLYSIVSQSVGPELGAAIGILLSIANTVSAIFNSWLLVSVLIYNFNAIDDTISTILIDYITLFRIVLIIFMSILCYIAMDNETRIQYALLITIIFGIFNILIGSCIGPKTNLEKASGFTGLNMATLKKNWYSDYRIEDHEQQHFFTVFATFFPCLTGIHAGVKYSSGDLEVPSTSIPKGTLLSILITTTSYILLTVIHGSVQLREASGNETELHDGSFTNCSFRYCDKGLRNEGYTTISLWPISIYFAYIAATISNIISTLIFVPKLLQIMGQDDVFPLLKYLAKGYGKNNRPFRSMILLLICIIIIGSFVKHSSHIWFMASFSTTCFLFAYVMLNLCTFHNAHFKPLYWRPSYKIYNKWISLAAAVICISLMIFINTKLSLIIASAVCVIYIIALKKRDSSMKIQQIKTIIRSLYKADTIKYHIKNYLPNLIVFSGNPKSRKKLVSLAHLITKNNGVQMCVSVEKISITPKQKKIYIDKGYQWLRSSRINSLYVVLDNLELDLATHMIYSCGHGQLRPNITMIGYKSDWLNCPYQDLQTYLNIFNVANMNDMSTIVVRVSSTESYDVQNLLIRNFNHLDHNDDISCETQSSQNIGNQQKTTPKDDEGINILFMCWMETITRGLPPCIIINGSTEQVLAVNS |
| *DnAPC14*  (*rna-XM_015515908.1*) | MFCSFLYSSLYSNDILNIINSNLSTGALGVVSIVIMSILGCIGMDIEAEVQYALLITIIVGIFNLIIGSYIRPKSDLEMAPDFTGYNMKNYLDYENFKYFLMIFTRFFPTVTSINAGVKYSPEDLKVMLYQYSIIIIILI |
| *DnAPC15*  (*rna-XM_015516217.1*) | MSELEEIKNKNRPWINQIQSFNKLYRYEDNNAYLSPGIKLGWVIGVLIPCLLSTWNVMLFIRIPMIVGNAGILHSFLIAILSLLIILITNCSLTAISTNGKTKEGGLYFIISRSIGPEFGTYIGIILAFANIVSAIHIAPLVAVVFAFINVIIGAYIGPPTPLLKAEFITRFNMKTFEKNLYPKYDNFENVHESFLTMFTKFFPFFTGIHSGLKYSAGDLKDPNVAIPKGTLFSIIITTTMYIVIIVLSGSVQLREAMNAKIFDDVSFANCPFGGCYEGFTTTLFYRISLSKFFIYIGVITASISNTLTALIFGSKLLKRIGQDDVLPLIKYLXKGYGKSNEPYRAHVFTIVVSSTLFCIKIKYGVNSGVSFKLITICNLIAYTMLNYCTFLIAYSEPLGWRPKYKFYNKWSSLAAAIICSSLMIIINLTMSLNVGCAVCVLYILARSKNEVINWGSFTQTQQLKIVIKNMYMADTIQYHIKNYLPSLIVFSGNPKSRKKLVSLANLITNNNGVQMCVNIEKVLLSPRQKKIYLDKGIQWLKTSGIKSLYVIIDNIELDLAIPLIFSCGHGQLKPNMAMVGYKSDWLSCPYQELQTYLNIFNEGKIHDISMIMVRVSSTEFNDSQNLFNRGCKHLDHNEDIPCETQSNQNIRNQQKLDNCSLKIKNEEFSFDSEKRKNGTVDLWWLYSDGGLSLIISSILKYSITWKNCKFRIFGVTNKIERLSEEKHKLKQLLSLYRIDFDYLDIILANITDPTTITFFNSLLEHVASRDNQFDDYNLQKDHIAETLFLRDLIELHSFNSDLIILTTPNNNNEEINILFMCWIETISRGLPPCIIINRSADPVLSIYA |
| *DnAPC16*  (*rna-XM_015516639.1*) | MLDTYTKNVYQKLSRKKTYVEETTGVEKDKFKRVLNVVDLTALGTGSTLGCGVYVLAGTVAKSVAGPAVVLSFILAATVSSLSGVCYAEFASRVPKAGSAYIYSYVAVGEFIAFVIGWNLLVEHTIGTAAVAKAMSNYLDSLLGDPQKRFMKKHFPIHMDFLGEYPDIASFLFIMCIALVVAWGVRKSSTLNNIFTTLNLVTVCTIIGSGLYFANISNWFIDKSDIPPGVNGGNGGFLPFGWTGMVAGAARCFYGFIGFDSIASTGEETKNPKKTIPLAIVLTLLNVTVAYASVASVLTLMWPYYDQDPNAPLPVIYENLGMPVLKHLVTGGAVFALFTTLIGCLFPIPRILYAMSSDGLLFSFLATINEKTKTPFIAAIICGVCAGLLSTIFNLEQLVDMASIGTLQSYMIVCVCVLILRYKNNNLYSRDSVGPKFYTISMWLNVSNANDTNRDTQYVSRVLISIFTVTACIFCICVVNWDSHKATGRLILSFIICLSVIILLVVMLMMNRLPQAIESLSFKVPFVPFVPCLSIVLNLYLMMELNFKTWIRFSVWLVIGLLIYAFYGLKHSIEGIKDQSNEIKEGKNEQKISN |
| *DnAPC17*  (*rna-XM_015516645.1*) | MRQADDDVMMKLRETLYQVMFRRKNENDIGDDHPDKKKLARVLNLLDLTALGVGSTLGVGVYVLAGNVARIEAGPAVVLSFVLAAFASALAGLCYAEFAARVPRAGSAYVYSYVGVGEFVAFVIGWNLILEYVIGTASVAKAFSNYIDALLDYPVKTTMTYLFPMNVSFLADYPDVLSFSLVLLLSIILSWGVRESTMINNVFTVVNLLTVVTVVVSGLFKVNLYNWNIPKLDIPKSAKGGEGGFMPFGWAGVTAGAAKCFYGFIGFDSVATTGEEAKKPKRDIPLAIILSLTIITFAYCCISSVLTLMWPYYDQDIDAPFPYVYDQLGWTTLKIIVSSGAIFAMFASLLASMFSMPRILMTMAQDGLMFSMFSIIHSRFKTPLLATLFSGLLAGIITAILNLEQLMNMMSIGTLLAYTIVCICVLMLRYRNDEDGDQLVINGPETSSGFFXVVEKYFNLSNIDNANKETERVATTITVLYICTSVLFSFVTVQKECVVTTHQWCDDDDNNTAAFNLGCVVNTSHATTPFEQGCVENSIAKYAXAILAIGLLLLLLLLTRQPQSNKKLSFKVPLVPLIPCISILMNIYLMMKLDLITWIRFSIWLTIGLFIYVLYGMNNSAEGLKQKGELNRSRSSSAKSIHQNSSVSL |
| *DnAPC18*  (*rna-XM_015516638.1*) | MMDDPWYKSTDSLTWALTRKKTDSDEPSKEKLNRVLTFFDLTALGTGSTLGCGVYVLAGAVAKSIAGPAVVLSFAIAAVVSAFSGLCYAEFAGRVPKAGSAYIYSYVAVGEFTAFVIGWNLLIEHLIGTASVAKAMSNYCDSLLGNPQRKYMTEYFPLHIGFLADYPDLASFVVIVVIALLVAWGVRESSFTNNIFTALNLITVCTVIITGFYKANYSNWSIPKSEIPPEANGGEGGFLPFGWVGVAAGAAKCFYGFIGFDSIATTGEETKNPKRDIPLAIVASLFLSTVAYCGVATVLTLMWPYYSQDPDAPLPALYENLDMPTIKIIVSGGAIFALCTSLLGAIFPLPRILYAMASDGLLFKFLSNINATTKTPLISTIICGVFAGSLAAVFNLEQLIDMASIGTLQAYTIVCICVLILRYTDNSPSIQDNTIKSKRITVFTWLNLSNAKVPNSDTQYVSRALIFIFSVCTFVFGISLASMESHLGNTRNVLMIINVISLLVLLITLFMLARLPMAVEDLSFKVPLVPIIPCLSIVLNVYLMMELEYKTWIRFIVWLICGLLIYLFYGIGHSLEGNKQKIMLNTIQIKPKLSS |
| *DnAPC19*  (*rna-XM_015516653.1*) | MRNLSTHTLYQTLCRKKTFNDEVEPGKEKLKRVLNIFDLTALGIGSTLGCGVYVLAGTVAKSIAGPAVVLSFIVAAIVSSFSGVCYAEFAGRVPKAGSAYIYSYVTVGEFIAFFIGWTLFIEHTIGTASVAKAMTNYLDALLGDPQKIYFKKHLPMHVDFLGEYPDFASFFFVIFIGLIVAWGVKKSSTLNKMFTLLNLLTLGTVVASGFFLGKLSNWFIPKSEIPLGMDGGNGGFSPFGWNGIIAGAARCFYGFIGFDSIATTGEETKDPKRTIPLAIILSLFFVTLAYSSVASVLTLMWPYYDQDPDAPLPVIYENMGMPIIKYMVTCGAVFALMTTLLGCLFPIPRILYAMSSDGLLFKCLSTVNEKTKTPVLATVFCGIGTGLLSCMFNLEQLVEMTSIGTLMSYLMVCVCILILRYKNNNTVNQNLDNSEVHIIYKWWSASNTGLTSMGSQYVSRVLIITYTFAAFVFCVCMTNVDYYEGPLQLLLTIVIGISVSIMLISLLMLCKLPQAIENLSFKVPLVPLIPCLSILLNLYLMMELNTKTWMRFGIGIVVGLLIYAFYGVHNSLEGSKRRTIKDKEYKQEIKISN |
| *DnAPC20*  (*rna-XM_015516636.1*) | MFYQTLSRKKSFTAETKQERDKLKRVLTIFDLTALGIGATLGSGVYVLAGTVAKSIAGPAVVLSFIVAAIVSSFAGVCYAEFAGRVPKAGSAYIYSYVAVGEFIAFIIGWNMFIEHTIGTASAAKAMTNYLDSLLXDPQKKYMKVHFPIHMKFMGEYPDVASFXFLMFIACIYQHLFIV |
| *DnAPC21*  (*rna-XM_015516789.1*) | MNIEKSKNVEITNSISRKKGDRVRLKKQLGLLEGVAIILGIIFGSGIFISPSGVMNEAGSVGVSLVVWLMCGMLSMIGALCYAELGTSIPRSGGDYTYLFEGYGPLPAFLYLWDAMLVFVPTTNAIMGLTFSNYVIKPFFAECDNPEQAVRLLAAAVICFITFINCWNVKATTKVQNVFMFTKIFALXLIIVCGVVYLYSNGFSKFINPWQGSVTDPGRLAVSVYSGIFSYSGWNYLNFMTGELRNPYVNLPRAIYISMPLVTIIYVLANVAYLAVLTPYDMVTTKAIAVTFGHLAMGKFKWIMPLMVSLSAFGGLCVNIMTSSRLCCVAARHGHFPMFLSYINVKRYTPIPSLVFLNILSLLMLFTTDVNKLITYSSIVEAFFTMLSVSLVLWNRWKRPNINRPIKVSLWIPITYVIVSLFLIALPCYVRSFEVGMGVGITLLGIPVYYICVVWKTKPEWFQNSLKHVTFTTQKLFVVAKEEKADNVLE |
| *DnAPC22*  (*rna-XM_015517820.1*) | MLRNLQTSHGFNNNNDTNDDDGGDGDGETAGNGKNMLARKLSTKGDTLYLERRVGLYSGVALIVGTMIGSGIFVSPTGLLIRTGSIGLSFVIWAACGAMSLLGALAYAELGTMNPSSGAEYAYLMDAFGPMPAFLFSWISTLILKPSQVAIICLSFAKYAVEAFVDECGSSDFVVKIVAVLSILIITYINCYSVNLATGVQNAFTAAKLVAVFVVVAGGVYKIMQGHTEHFRNYFENTTTSVGDVATAFYSGLWAYDGWNNLNYVTEEIKNPSKNIPKAIYISIPLVTMCYLLVNVAYLTIMSPDEIVRNEAVAVTFGIRALGSIAWVIPLSITISTFGSANGTLFAAGRLCFAASREGHLMHVLSYIHIKKLTPMPSVIFHSIITIVMVASGTINSLIDFFSFTAWIFYGSAMLALLVMRYTRPDVPRPYKVPIIIPLTIFVISLYLVVAPIIDKPQIEYLYSVMFMIAGMIFYVPFVRLGYKFRIIDRWTVIIQLLLQVAPTKLVLPE |
| *DnAPC23*  (*rna-XM_015518051.1*) | MPPLPVQHQQQIDEGSRSSPARQHQNGGSPATMAAVVPSPNTPRKRGDVKDETYSSENNRNSGYETNLYLYSDEMDDRPRVPTFLNSIADYSNTIPSLAESDTKSTTAKQGSSRMGTLVGVYLPCVQNIFGVILFIRLSWVVGTAGVIYGFGIVFTCCCVTMLTAISMSAIATNGVVPAGGPYFLISRSLGPECGGAVGMLFYTGITLAASMYIIGAVEIILTYMAPSLSIFGDFSKDNSIMYNNFRVYGTILLMVMSSIVYVGVKFVNKFASVALACVLLSILSVYVGIFYNFHGSDKLYMCSLGNRLLKDMDLKYCNKAVGGYLYNMYCSNGTGACEDYFVRHNTSIVKGIRGISSGVFFENIFGNFLEKGQYIARGHLQEDILASSQNSYNMIMVDITTSFTILIGIFFPSVTGIMAGSNRSGDLADAQKSIPIGTILAILSTSSVYLSAVLLFGATVDNLLLRDKFGQSIGGRLVVANIAWPNEWVILVGATLSTLGAGLQSLTGAPRLLQAIAKDDIIPFLQPFAKSSASGEPTRALLITVAVCQCGILIGNIDNLAPLLAMFLLMCYAFVNLACVLQTLLRTPNWRPRFKYYHWTLSFTGLVLCIAVMFMSSWYFALLALGMAGIIYKYIEYRGAEKEWGDGMRGLALSAARYSLLRLEDAPPHTKNWRPQILMLVNFNADLPKYRKIFSLVSQLKAGKGLTVSATVIEGDFIKKTGHDVQSTKKELVRLMDEEKVKGFADVLVSNNTSEGLSHLIQIAGLGGLKPNTVILGWPNSWQQSENDRSWQVFLHTIRIVTAAKMALIVPKGIRSFPDSATKLSGTIDIWWIVHDGGILMLIPFLLKQHRTWKNCKLRIFTVAQTDDNSIQMKKDLKTFLYQLRIPAEVEVVEMTNNDISAYTYERTLMMEQRNQMLRELRLNKKESFGMMRNIIDFNRETSTGENTPLVQSIIDQHHRKDASEGRSATKVRFQEETSMDDEAKKSKSAESAFDVEDKRTTGNGDKPGVLKDSLPKETTDSSTSTMRPDEDNVRRMHTAVKLNEVIVSRSRDAQLVIFNLPGPPKDTKLERESNYMEFLEVMTEGLDKVLMVRGGGREVITIYS |
| *DnAPC24*  (*rna-XM_015520511.1*) | MTIDNVTAKLDNMDINCENTKSDNINIDCTIITETDGMKIGGVKTKSMSLNMLEKSESYDIFKQLPLEIINKIVLQLDFNDILNLKLVNKRWRCVYLNQDEIWAKVCEDLNIRAIDYNRCLNDRLKHDSECIGYAEITSEKLFGPLCNHWITFNHYLMLLKSIKSNDFPTIYIPRPYVEQSYCTDDYIININRYHRQPIQIVTLNGPHKPLNKRILPMFPKLKELIKLKEYPLKVIGNKRYLVIEICSIIFVYTIKNSAFDKRFFKVIQKAVNYGLDENDFNKDFLNNHCDTKFDLYDYKLVVVHPAISTLFLIDLKTEKTYKELQFSSRGCIVNSIKCSDYRLMIGITKKNENDLSKEHLAIVYHLKGKTQDNGLXICLLGPVTQFKVTKNCIGVENTGYSNVVTPFILKQNDLHLDVFWLKCDTFSFDSTRNYIYYNFNQSIFQFDLKAITSLQYEGIQKISNDSILSLLPLTPINDRYLLVKLAYHNSYEIFDVKERVYVRSIQLTSGYSLVHVGKLSIVFSNSSGYMVISFN |
| *DnAPC25*  (*rna-XM_015520736.1*) | MNSESPASIGVSFAGNIGEKIPLMNRRRQMFDYFTRMFGNSSTGSEETSKDGYVEFGNIGEATSGRTLGPFAGVFAPVCLSMFSAMLFLRVGFVVGNAGLIETLFQFAIAYTIIVFTVMSVCAISTNGAVEGGGAYFMISRTLGPELGGSIGTLFFLANIVSSALCLTGCVEGLIDNFGESGMLNFQLIVPSFKYFSAWTCSFGLMGSVIMMFVINPFYASTSFGLCLLLILLLHFFSPCEASEWGSISQALIFHQVRKYLLLLDPRKNHIKFWRPQMLLMVANPRSSCPTITFINDLKKSGLFVLGHVKVDKAETNLDYTDKTISSWLTLVDHVKVKAFVELTVANSVREGLHHLVRLSGMGAMKPNTVVLGFYDNKVPLDYFTRLDSVYKTSLFQGLTTNEEKFPLRLPNEEKHLSTSEFVSMICDIFQMHKNICICRHFDLYNKTDIVKNIKYKYIDVWPVNFFGPSMNDAYDTSSLFMLQLSCIIKMIAVYKKHILRVHLLNTIDIQSQRVQLKQLLSTLRIKASIHEVTEWSQIDLTTLDSDVNTYISRVNKLIRNQCAETIYVIK |
| *DnAPC26*  (*rna-XM_015520786.1*) | MVRLRETLYQVIFRRKKEDDIEVDQSDKKKLSRVLNLVDLTALGVGSTLGVGVYVLPGNVARXDAGPAVVMSFVLAALASVVAGLCYAEFAARVPRAGSAYVYSYVGVGEFVAYVIGWNLILEYVIGTASVAKALSNYIDALLDYPIKSTMSSLFPMDVSFLAEYPDVLSLSLVLLLSVILSWGVRESTMINNVFTVVNLLTVVIVVVSGLFKINLYNWSVPKQDIPRNAKGGEGGFMPFGWAGVTTGAAKCFYGFIGFDSIATTGEEAKKPKRDIPLAIXLSLIIITFAYCCISSVLTLLWPYYDQDIDAPFPYVYDQLGWTTLNIIVSFGAIFAMFASLLASMFSMPRILMTMAEDGLMFSMFSIIHPKLKTPLFATLMSGVFAGIITALLNLDQLMNMMSIGTLLAYTIVCICVLMLRYRNDFDGGVFVENLGNDEPETSGFVKMVEKFCNLSKIDNTNKDTERVATTITVLYICVSALFSFVTIQQECDVIIYPWCDESDSNAATFQSGCVINTYNATTPFEQPCVANNITKYTSVILAIGLLLLLLLLSRQPQSRKKLSFKVPLVPLIPCISILMNVYLMMKLDMITWIRFSIWLVIGLFIYALYGMNNSVEGLKQKRQLKINSPRSAEPIHELYTSYL |
| *DnAPC27*  (*rna-XM_015521357.1*) | MFAGLCYAEFAARVPKAGSAYVYSYVGVGEFVAFVIGWNLILEYVIGTASVAKGLSNYIDALLEYPMKRTMTDLFPMNISFLSEYPDFLSFSVVLLLSILLSWGVRESTMINNVFTVVNLLTVATVVITGLFKVNSYNWNIPKQDIPKTAKGGEGXFMPFGWAGVTAGAAKCFYGFIGFDTVATTDDHLGWTTIKWIVSSGAIFALFTSLIGTMFPLPRILYAMSCDGLLFSMFSDIHPRYQTPVLATLLSGMFAGIMSAIFNLEQLIDMMSIGTLLAYSIVCICVLVLRYRNDSDVEFVIKGNDESETSGFFETVVKTVVRYFNLSNIKYANEATESVATIITMWFICTSALFCFITVQQESAENSSDVVVYSSAILAIGLLLLLLLLARQPQSTKELSFKVPLVPLIPCISILLNIYLMMKLDIHTWIRFGIWLLIGLFIYVLYGMKHSVEGRKQLGEPKKRPSTAAPVHPISIIKL |
| *DnAPC28*  (*rna-XM_015522424.1*) | IQSFSGLYHRGTENKVEVSPGIKLGWIKGVTIPCLLSTWSVMIFYKMPWTLGQAGILISIIIIFLSMLIILFTNFSLSVISTNGKIKGGGLYSIVSQSVGPELGVVIGMLLALANTVSAMLNSWLLVIILEEIDDTIFTILIDYIILLRIVLIIFLSILCYIVMDNESKIQYALLITIIFGIFNVLIGSCIGPKSNLEKASGFTGFNMATLKKNWYSDYRIFRHEQTNFFTVFAIFFPCLTGIHAGVKYSSGDLEVPSTSIPKGTLLSILITTTTYILLTVIPGSVQLREASGNETELHDGSFTNCSYRNCYGGLSHDVDI |

Supplementary Table 3. TPM values of APC transporter genes of BPH at different developmental stages. Stage-specific gene expression level of APC transporters was calculated using Kallisto v0.46.1 based on a series of RNA-seq data downloaded from NCBI database. A total of 15 different developmental stages were evaluated in this study.

| **Gene** | **egg24h** | **egg48h** | **Egg5D** | **1st24h** | **1st48h** | **2nd24h** | **2nd48h** | **3rd24h** | **3rd48h** | **4th24h** | **4th48h** | **5th24h** | **5th48h** | **A24h** | **A72h** |
| --- | --- | --- | --- | --- | --- | --- | --- | --- | --- | --- | --- | --- | --- | --- | --- |
| *NlAPC01* | 0.15 | 0.11 | 29.83 | 23.32 | 27.00 | 26.17 | 19.86 | 17.43 | 15.47 | 7.42 | 6.63 | 9.46 | 7.99 | 12.17 | 9.88 |
| *NlAPC02* | 0.19 | 0.09 | 0.21 | 0.19 | 0.18 | 0.27 | 0.19 | 0.28 | 0.31 | 0.31 | 0.28 | 0.34 | 0.55 | 0.52 | 0.70 |
| *NlAPC03* | 8.80 | 8.01 | 21.20 | 11.69 | 12.10 | 18.41 | 8.62 | 18.26 | 12.35 | 11.17 | 12.25 | 8.37 | 13.64 | 10.10 | 10.68 |
| *NlAPC04* | 0.91 | 0.10 | 0.34 | 3.11 | 1.45 | 3.10 | 2.65 | 1.78 | 2.65 | 0.76 | 0.51 | 3.33 | 3.16 | 1.00 | 2.64 |
| *NlAPC05* | 0.00 | 0.02 | 4.14 | 7.34 | 5.56 | 5.69 | 5.51 | 4.01 | 3.79 | 1.92 | 1.79 | 2.27 | 1.90 | 0.56 | 0.44 |
| *NlAPC06* | 0.15 | 0.08 | 0.21 | 0.17 | 0.25 | 0.15 | 0.27 | 0.07 | 0.08 | 0.26 | 0.26 | 0.30 | 0.16 | 0.09 | 0.26 |
| *NlAPC07* | 12.97 | 12.80 | 13.75 | 16.12 | 9.38 | 7.73 | 9.20 | 7.44 | 5.07 | 7.05 | 3.20 | 4.93 | 6.67 | 17.82 | 24.61 |
| *NlAPC08* | 0.00 | 0.10 | 0.00 | 0.00 | 0.00 | 0.00 | 0.12 | 0.00 | 0.00 | 0.13 | 0.00 | 0.00 | 0.14 | 0.00 | 0.11 |
| *NlAPC09* | 2.60 | 4.74 | 11.69 | 11.22 | 8.79 | 6.56 | 7.90 | 8.22 | 4.88 | 10.21 | 9.66 | 9.87 | 7.41 | 12.04 | 18.69 |
| *NlAPC10* | 2.37 | 2.29 | 20.50 | 27.95 | 29.11 | 21.86 | 24.74 | 22.37 | 24.72 | 21.99 | 15.12 | 25.05 | 22.08 | 38.28 | 28.67 |
| *NlAPC11* | 16.31 | 9.45 | 7.73 | 31.67 | 26.31 | 18.45 | 26.01 | 23.47 | 18.48 | 28.36 | 23.82 | 28.95 | 29.86 | 43.18 | 31.13 |
| *NlAPC12* | 16.35 | 11.53 | 8.54 | 17.36 | 12.64 | 9.74 | 8.43 | 11.43 | 6.94 | 10.36 | 6.15 | 9.26 | 13.57 | 12.17 | 10.10 |
| *NlAPC13* | 3.36 | 5.41 | 9.00 | 9.25 | 16.68 | 10.31 | 16.89 | 10.55 | 13.96 | 7.98 | 5.34 | 9.39 | 9.11 | 13.12 | 7.98 |
| *NlAPC14* | 0.05 | 0.02 | 5.99 | 8.91 | 8.13 | 5.95 | 5.98 | 5.12 | 4.21 | 3.25 | 1.70 | 2.57 | 2.29 | 1.34 | 0.93 |
| *NlAPC15* | 1.46 | 1.19 | 2.86 | 3.24 | 3.36 | 2.96 | 2.80 | 2.33 | 3.05 | 2.67 | 2.14 | 2.65 | 3.46 | 3.00 | 3.11 |
| *NlAPC16* | 0.12 | 0.18 | 1.19 | 22.79 | 21.67 | 22.51 | 21.41 | 22.43 | 22.08 | 43.57 | 22.07 | 46.29 | 33.20 | 37.36 | 30.11 |
| *NlAPC17* | 34.65 | 19.61 | 21.23 | 45.23 | 33.21 | 38.98 | 24.13 | 41.30 | 26.67 | 35.97 | 26.48 | 31.20 | 30.79 | 38.68 | 39.18 |
| *NlAPC18* | 0.00 | 0.00 | 2.68 | 5.66 | 4.77 | 3.34 | 3.04 | 2.51 | 1.82 | 1.56 | 1.28 | 1.61 | 1.15 | 0.41 | 0.29 |
| *NlAPC19* | 3.16 | 6.61 | 18.22 | 15.83 | 33.71 | 29.01 | 30.59 | 22.00 | 34.05 | 15.59 | 21.16 | 12.26 | 15.31 | 29.44 | 19.48 |
| *NlAPC20* | 0.00 | 0.00 | 0.14 | 0.17 | 0.54 | 0.19 | 0.46 | 0.25 | 0.25 | 0.06 | 0.00 | 0.06 | 0.00 | 0.05 | 0.00 |

Supplementary Table 4. TPM values of APC transporter genes of BPH at different tissues. Tissue-specific gene expression level of APC transporters was calculated using Kallisto v0.46.1 based on transcriptome data obtained from NCBI database. A total of 6 different tissues were evaluated in this study.

| **Gene** | **Head** | **Salivary gland** | **Integument** | **Gut** | **Ovray** |
| --- | --- | --- | --- | --- | --- |
| *NlAPC01* | 1.30 | 0.31 | 6.46 | 5.04 | 0.12 |
| *NlAPC02* | 0.03 | 0.04 | 0.14 | 0.24 | 0.80 |
| *NlAPC03* | 3.81 | 0.91 | 6.53 | 6.38 | 11.59 |
| *NlAPC04* | 0.09 | 0.05 | 0.26 | 0.04 | 4.48 |
| *NlAPC05* | 0.00 | 0.07 | 0.00 | 0.00 | 0.00 |
| *NlAPC06* | 0.41 | 0.06 | 0.05 | 0.04 | 0.06 |
| *NlAPC07* | 2.48 | 5.15 | 14.78 | 22.35 | 18.13 |
| *NlAPC08* | 0.00 | 0.00 | 0.00 | 0.00 | 0.00 |
| *NlAPC09* | 13.62 | 5.96 | 13.96 | 28.19 | 26.81 |
| *NlAPC10* | 3.60 | 1.14 | 20.27 | 0.62 | 4.18 |
| *NlAPC11* | 8.57 | 7.69 | 39.28 | 337.70 | 4.05 |
| *NlAPC12* | 0.73 | 0.39 | 2.43 | 0.54 | 11.95 |
| *NlAPC13* | 4.51 | 0.54 | 8.92 | 32.19 | 0.94 |
| *NlAPC14* | 1.43 | 0.45 | 0.11 | 0.00 | 0.05 |
| *NlAPC15* | 0.64 | 0.24 | 0.97 | 1.44 | 3.43 |
| *NlAPC16* | 9.88 | 5.03 | 3.89 | 892.80 | 4.99 |
| *NlAPC17* | 22.28 | 0.40 | 12.84 | 587.84 | 5.61 |
| *NlAPC18* | 0.12 | 0.08 | 0.02 | 0.00 | 0.02 |
| *NlAPC19* | 11.83 | 4.72 | 37.56 | 39.95 | 10.85 |
| *NlAPC20* | 0.00 | 0.00 | 0.00 | 0.00 | 0.00 |
